# Corrected placement of *Mus*-*Rattus* fossil calibration forces precision in the molecular tree of rodents

**DOI:** 10.1038/srep14444

**Published:** 2015-09-28

**Authors:** Yuri Kimura, Melissa T. R. Hawkins, Molly M. McDonough, Louis L. Jacobs, Lawrence J. Flynn

**Affiliations:** 1 Department of Geology and Paleontology, National Museum of Nature and Science, 4-1-1 Amakubo, Tsukuba, Ibaraki 305-0005, Japan; 2 Department of Vertebrate Zoology, National Museum of Natural History, NHB 390, MRC 108, Smithsonian Institution, P.O. Box 37012, Washington, DC 20013-7012, USA; 3 Center for Conservation and Evolutionary Genetics, Smithsonian Conservation Biology Institute, National Zoological Park, Washington, DC 20008, USA; 4 Roy M. Huffington Department of Earth Sciences, Southern Methodist University, Dallas, Texas 75275, USA; 5 Peabody Museum and Department of Human Evolutionary Biology, Harvard University, Cambridge, Massachusetts 02138, USA

## Abstract

Time calibration derived from the fossil record is essential for molecular phylogenetic and evolutionary studies. Fossil mice and rats, discovered in the Siwalik Group of Pakistan, have served as one of the best-known fossil calibration points in molecular phylogenic studies. Although these fossils have been widely used as the 12 Ma date for the *Mus*/*Rattus* split or a more basal split, conclusive paleontological evidence for the nodal assignments has been absent. This study analyzes newly recognized characters that demonstrate lineage separation in the fossil record of Siwalik murines and examines the most reasonable nodal placement of the diverging lineages in a molecular phylogenetic tree by ancestral state reconstruction. Our specimen-based approach strongly indicates that Siwalik murines of the *Karnimata* clade are fossil members of the Arvicanthini-Otomyini-Millardini clade, which excludes *Rattus* and its relatives. Combining the new interpretation with the widely accepted hypothesis that the *Progonomys* clade includes *Mus*, the lineage separation event in the Siwalik fossil record represents the *Mus*/*Arvicanthis* split. Our test analysis on Bayesian age estimates shows that this new calibration point provides more accurate estimates of murine divergence than previous applications. Thus, we define this fossil calibration point and refine two other fossil-based points for molecular dating.

Divergence time estimates in phylogenetic studies have become increasingly valuable for addressing questions regarding lineage diversification rates, evolutionary patterns, and historical biogeography, among others^1^,^2^. Fossil data are the most common type of calibration used to estimate absolute node ages for trees. However, inherent problems exist as a source of error when incorporating fossil priors for calibrating trees, including completeness of the fossil record, age accuracy of fossils or strata, and correct phylogenetic placement of fossil calibration^3^,^4^,^5^,^6^. In this study, we use a specimen-based approach to show that one of the most widely-used fossil dates for mammal phylogenies, the *Mus*/*Rattus* split (mice/rats divergence)^7^,^8^,^9^, had been placed at the incorrect node.

The paleontological event on which this datum (~12 Ma) is based is the first appearance of the fossil genus *Progonomys* in geologic time^10^,^11^,^12^. This event evolutionarily represents the first split from the earliest definitive murine, *Antemus*, and the acquisition of derived dental characters of crown Murinae (connections of anterostyle and enterostyle with corresponding medial cusps)^10^,^11^,^12^. Owing to frequent citations of Jacobs and Downs^10^, fossil murine rodents from the Siwalik Group of Pakistan are among the most prominent paleontological records utilized as fossil calibration (Fig. S1). As paleontologists suggested^10^,^11^,^12^, the Siwalik fossil-based date was widely accepted as the *Mus*/*Rattus* split in molecular phylogenetic studies in the 90’s and early 2000’s^13^,^14^,^15^,^16^,^17^,^18^,^19^,^20^,^21^,^22^. More recently, molecular phylogenetic studies have clarified evolutionary relationships of the Murinae and confirmed that the earliest split within the subfamily is the divergence between the Phloeomyini and core Murinae, followed by the *Mus*/*Rattus* split within core Murinae^9^,^22^,^23^,^24^,^25^,^26^,^27^,^28^,^29^,^30^. As a result, this 12 Ma fossil-based date is now more commonly assigned to the *Phloeomys*/core Murinae split^9^,^24^,^26^,^27^,^29^,^30^,^31^. However, accepting the transition from *Antemus* to *Progonomys* as a calibration point for the *Phloeomys*/core Murinae split is entirely dependent on molecular tree topology, and direct morphological evidence has not been demonstrated in the fossil record. None of the Siwalik specimens possess derived dental characters (a cordate-shaped anteroconid on lower first molars and a well-developed posterostyle on upper first molars) shared by members of the Phloeomyini^32^.

The rationale for considering the first appearance of *Progonomys* as the *Mus*/*Rattus* split is based on the simplified phylogenetic hypothesis of Siwalik murine rodents by Jacobs^33^ and Jacobs and Downs^10^, which proposed dichotomous lineages (the *Progonomys* clade and the *Karnimata* clade), deriving from *Antemus* (Fig. 1A, see SI Discussion for details). *Mus* was confidently placed in the *Progonomys* clade based on observation of gradual morphological change from older *Progonomys* to younger *Mus auctor* in Siwalik murines^10^,^33^,^34^. On the other hand, *Rattus* was placed in the *Karnimata* clade with some uncertainty^10^, along with extant genera of the Arvicanthini (*Arvicanthis*, *Pelomys*, *Mylomys*, and *Golunda*), heavily relying on the anterior displacement of the anterostyle^10^,^33^,^34^ (dental terminology in Fig. 1B). No further morphological evidence is found for the evolutionary relationship between *Rattus* and Siwalik species of the *Karnimata* clade^35^.

In more recent studies, we have shown that diverging tooth morphology in the Siwalik lineages was initiated by 11.2 Ma, with decreasing morphological overlap through time^34^ between the *Progonomys* and *Karnimata* clades (Fig. 1C), and that the 11.2 Ma stratigraphic occurrence can be considered as the evidence of lineage separation under the initial split criterion^36^. In this study, we examine the most reasonable placement of this separation event in a molecular phylogenetic tree by presenting new qualitative characters that define the lineage separation and tracing ancestral states of the characters in a molecular phylogenetic tree of extant species.

Here we show that progressive acquisition of new dental characters in the *Karnimata* clade is strong evidence for Siwalik murine rodents representing a younger node, the *Mus*/*Arvicanthis* split, than previous applications. The finding of this study constrains the timing of the *Mus*/*Arvicanthis* split to ~11.2 Ma as a stratigraphic occurrence and to 11.36 Ma by a molecular divergence estimate and further allows revision of calibration dates for two additional clades. These fossil-based dates are exceptionally highly resolved, compared to any other estimate thus far, because the divergence event is morphologically tracked in geologic time and because of well-constrained age estimates of Siwalik localities within both biostratigraphic and paleomagnetic frameworks.

## Results

Frequency distributions of the size and inclination of the metacone (Fig. 2A–E) and the presence of the posterostyle were evaluated for each Siwalik species (Fig. 3, Tables S1–S3, Dataset S1). A large metacone that is posteriorly inclined parallel to the paracone (Fig. 2A) is plesiomorphic for the Murinae. In the *Karnimata* clade, ?*Karnimata* conserves this plesiomorphic condition in 60% of the specimens at 11.2 Ma (Table S2). By 8.2 Ma, this plesiomorphic condition disappears from the *Karnimata* clade. Instead, a new combination of character states, a small metacone in vertical orientation (Fig. 2E), appears for the first time in 24% of *Karnimata darwini* at 9.2 Ma (Fig. 3, Table S2). The frequency of the derived condition (small, vertical) exceeds half of the specimens at 8.8 Ma and reaches 85% by 7.4 Ma. In contrast, the plesiomorphic condition of the metacone persisted in the *Progonomys* clade (Fig. 3). The frequency of the plesiomorphic condition continuously increases as the frequency of the ‘large, slightly inclined’ state decreases through time. By 8.8 Ma, more than 90% of the specimens have this plesiomorphic condition. None of the specimens in the *Progonomys* clade have the derived condition of the metacone (Table S3). As expected from the observations in each clade, the correlation between the two characters (size, inclination) is stronger in the *Karnimata* clade (Goodman-Kruskal’s gamma = −0.76) than in the *Progonomys* clade (Goodman-Kruskal’s gamma = −0.39). The null hypothesis of independence between the variables was rejected for the *Karnimata* clade (*M*^*2*^ = 23.9, *df* = 2, *p* < 0.0001) but was accepted for the *Progonomys* clade (*M*^*2*^ = 1.92, *df* = 2, *p* = 0.38).

The analysis of ancestral state reconstruction shows that a combination of the ‘small’ and ‘vertical’ states of the metacone is concentrated in the Arvicanthini (Fig. 4A, Fig. S2 for black-and-white). Maximum likelihood unambiguously placed it as the most probable ancestral state (93.0%) of the metacone for the Arvicanthini. In the node of the Arvicanthini and Otomyini, a small metacone in vertical orientation is predicted as the most probable ancestral state (80.3%) but is marginally insignificant over the second most probable ancestral state, a large metacone in parallel orientation (12.2%, difference of log likelihood = 1.89). For the node of the Arvicanthini, Otomyini, and Millardini (hereafter the Arvicanthini-Otomyini-Millardini clade), prediction for vertical orientation of the metacone (71.9%) is greater than that for parallel orientation (26.3%).

## Discussion

Siwalik murines of the *Karnimata* clade demonstrate progressive acquisition of a derived condition of the metacone (a combination of small size and vertical orientation Fig. 2E), which appeared as a minor variation at 9.2 Ma and became a dominant character state by 8.8 Ma. In contrast, this condition does not occur in any individuals of the Siwalik *Progonomys* clade. Ancestral state reconstruction suggests that a small meta-cone in vertical orientation is a synapomorphic character for the Arvicanthini-Otomyini-Millardini clade (Fig. 4A). Inside that clade, three arvicanthine genera (*Golunda*, *Mylomys*, *Stochomys*) and the Otomyini secondarily lost the character in acquiring their specialized tooth morphologies (Fig. 4A, see SI Discussion for details and Fig. S3 for photographs). Outside the Arvicanthini-Otomyini-Millardini clade, a vertical metacone was independently acquired by five genera, associated with the development of the posterostyle (Fig. 4A, see SI Discussion for details and Fig. S3 for photographs). In contrast, all genera of the Arvicanthini-Otomyini-Millardini clade (except *Thamnomys* and *Grammomys*) and Siwalik species of the *Karnimata* clade lack the posterostyle. Thus, based on the increased frequency of the derived condition of the metacone in the *Karnimata* clade (Fig. 3) and a continuous absence of the posterostyle, Siwalik species of the *Karnimata* clade are placed definitively as members of the Arvicanthini-Otomyini-Millardini clade. Importantly, the possibility of close relationships between the *Karnimata* clade and the Rattini is rejected. Combining the new observations and the widely-accepted hypothesis that the *Progonomys* clade includes *Mus*^10^,^11^,^33^, it is most logical to conclude that the progressive morphological divergence between the *Progonomys* and *Karnimata* clades represents the *Mus*/*Arvicanthis* split, which is more internal than the *Mus*/*Rattus* split at the tribal level. The independent acquisition of a vertically-oriented small metacone in the Apodemini within the *Progonomys* clade (Fig. 4A) is consistent with the paleontological hypothesis that European species of *Progonomys* is ancestral to *Apodemus*^37^.

Absolute nodal ages were estimated with the 11.2 Ma fossil date of the *Mus*/*Arvicanthis* split using the published data of Fabre *et al.*^29^ (Figs 4B and S4) and were then compared with the results of Fabre *et al.*^29^ (Tables S4). In three separate analyses of Fabre *et al.*^29^, the 12 Ma fossil date (i.e., the first appearance of *Progonomys*) was applied to two older nodes (the *Mus*/*Rattus* split and the *Phloeomys*/core Murinae split, respectively) and was excluded from analysis for cross-validation of the fossil constraint. Newly calibrated ages in this study are consistently older than the three applications of Fabre *et al.*^29^, and our molecular estimates are more congruent with paleontological evidence compared to any previous studies^9^,^22^,^26^,^29^,^30^ (Table S4). For example, the posterior mean age for the *Phloeomys*/core Murinae split (i.e., the node of the crown Murinae) estimated to be 13.6 Ma matches with the fossil evidence (13.8 Ma) of the first definitive murine rodents, which is outside the 95% CI of Fabre *et al.*^29^ (Fig. 4). This new calibration point for the most diverse group of modern mammals is unique in that a lineage separation event is identified based on morphological divergence, rather than the stratigraphic occurrence of the most basal taxa, and that the rich fossil record within a magnetostratigraphic framework constrains the bounding of the molecular divergence dating.

Based on our results, we explicitly define the lineage separation event of the Siwalik murines as a fossil calibration point of the *Mus*/*Arvicanthis* split and newly refine two fossil-dates for the Arvicanthini-Otomyini-Millardini clade and for the Murini, using Siwalik fossils. We follow Parham *et al.*^38^, who proposed a standard system for introducing new fossil calibrations, including a series of steps with an emphasis on the use of museum specimens to clarify the phylogenetic position of the fossil calibration.

## The *Mus*/*Arvicanthis* split

**Referred Specimens —** M1 specimens from localities Y791 and Y797, which are assigned to ?*Karnimata*.

**Figure 1:**
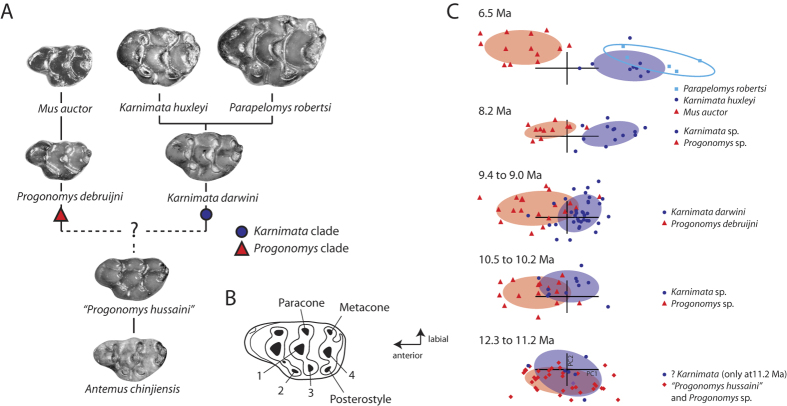
Phylogenetic hypothesis, dental terminology, and lineage separation in Siwalik Murinae. (**A**) Simplified phylogenetic hypothesis for Siwalik murine rodents proposed by Jacobs^33^ and Jacobs and Downs^10^ (after Kimura *et al.*^34^). (**B**) Dental terminology used in this study. 1. protocone, 2. anterostyle, 3. enterostyle, 4. hypocone. (**C**) Quantitative assessment of lineage separation in Siwalik murine rodents using geometric morphometric analysis on tooth outline of the upper first molars (details are found in Kimura *et al.*^34^).

**Paleontological Event —** The first appearance of ?*Karnimata*, interpreted to be close to the initiation of lineage separation between the *Progonomys* and *Karnimata* clades^36^ (Fig. 1C).

**Stratigraphic Occurrence (Age) —** Localities Y791 and Y797 (median: 11.2 Ma); Nagri Formation, Siwalik Group, Potwar Plateau, northern Pakistan.

**Age Determination —** Magnetic polarity stratigraphy correlated to the Geomagnetic Polarity Time Scale of Ogg and Smith^39^.

**Minimum Age Constraints —** 11.1 Ma; the upper ages of Y791 and Y797, associated with the first appearance of ?*Karnimata.*

**Maximum Age Constraints —** 12.3 Ma; the lower age of locality Y634 (median: 12.3 Ma), associated with the first appearance of *Progonomys*.

**Reference —** This study and Kimura *et al.*^36^.

## Most recent common ancestor of the Arvicanthini, Otomyini, and Millardini

**Referred Specimen —** M1 specimens of *Karnimata darwini* from Y182 that have a small metacone in vertical orientation.

**Paleontological Event —** The first occurrence of the derived condition (small, vertical) of the metacone in a chronoclinal assemblage of the *Karnimata* clade (Fig. 3).

**Stratigraphic Occurrence (Age) —** Locality Y182 (median: 9.2 Ma); Dhok Pathan Formation, Siwalik Group, Potwar Plateau, northern Pakistan.

**Age Determination —** Magnetic polarity stratigraphy correlated to the Geomagnetic Polarity Time Scale of Ogg and Smith^39^.

**Minimum Age Constraints —** 8.7 Ma; the upper age of Y388 (median: 8.8 Ma), associated with >50% frequency occurrence of the derived condition of the metacone in *Karnimata* sp. (Fig. 3).

**Maximum Age Constraints —** 10.1 Ma; the lower age of locality Y311 (median: 10.1 Ma), which is stratigraphically the youngest locality with no occurrence of the derived condition of the metacone (Fig. 3).

**Reference —** This study.

## Most recent common ancestor of the Murini

**Referred Specimen —** Lower m1 specimen of *Mus* sp. from Y547.

**Paleontological Event —** The first appearance of the genus *Mus* in geologic time.

**Stratigraphic Occurrence (Age) —** Locality Y547 (median: 8.0 Ma); Dhok Pathan Formation, Siwalik Group, Potwar Plateau, northern Pakistan.

**Age Determination —** Magnetic polarity stratigraphy correlated to the Geomagnetic Polarity Time Scale of Ogg and Smith^39^.

**Minimum Age Constraints —** 7.3 Ma; the upper age of Y931 (median: 7.4 Ma), which yields more *Mus* specimens than Y547.

**Maximum Age Constraints —** 8.3 Ma; the lower age of locality Y24 (median: 8.2 Ma), which is stratigraphically the youngest locality with abundant murine specimens, yet lacking *Mus* specimens.

**Other note —** Stable isotope values are associated with the specimen^40^.

**Reference —** This study and Kimura *et al.*^40^.

Paleomagnetic and biostratigraphic studies in the Siwalik group designate each small mammal fossil locality as belonging to a chronologically controlled stratigraphic bin of 100,000 years maximum duration (see SI Discussion for details). The bins were constructed by subdividing stratigraphic intervals between magnetochron boundaries^41^,^42^. This dating precision to ≤100,000 years, in combination with the fine-scale fossil record, is the great strength of Siwalik Muridae for molecular clock calibration studies, providing narrow intervals for the new fossil calibrations.

While the usage of molecular divergence dating is vast, *a priori* evaluation of fossil calibration quality is crucial for accurate Bayesian estimates of divergence times^2^,^3^,^4^,^5^,^6^. A comparative simulation study shows that an *a posteriori* cross-validation approach may select highly precise but inaccurate calibration points that are not consistent with fossil evidence^5^. Our results better corroborate paleontological estimates and greatly reduce the range of the prior age for the calibration point from the 95% credible interval of ~10 million years^29^, an interval nearly equaling to most of Murine evolutionary history (~14 million years), to that of ~2 million years. We further emphasize the fundamental importance of a well-dated and documented fossil record for evolutionary questions concerning modern organisms that rely on molecular time estimates. Such questions include but are not limited to diversification rates, evolutionary patterns, and biogeographic events, which are influenced by geological processes and climate changes through time.

## Methods

Fossil specimens (n = 272) of upper first molars (M1), ranging in age from 14.3 to 6.5 Ma, were examined in this study (Dataset S1). They were collected from the Potwar Plateau, northern Pakistan, in the 1970’s to 2000, and are currently on long-term loan from the Geological Survey of Pakistan at the Peabody Museum of Archaeology and Ethnology, Harvard University. We follow Kimura *et al.*^36^ for systematic classification of Siwalik murines. Ages of the Siwalik localities derived from paleomagnetic stratigraphy are based on Geomagnetic Polarity Time Scale 2004^43^. Modern specimens (n = 500) examined in this study (Dataset S2) include individuals of 70 genera and 79 species, comprising 54% (70/130) of the total genera of Murinae and 79% (31/39) of the genera within the *Mus*/*Arvicanthis* split^22^,^29^,^44^. These specimens are curated in the Museum of Comparative Zoology (MCZ), Harvard University, and in the Smithsonian Institution National Museum Natural History (USNM). Digital images of upper molars of these species are provided in Fig. S3.

**Figure 2:**
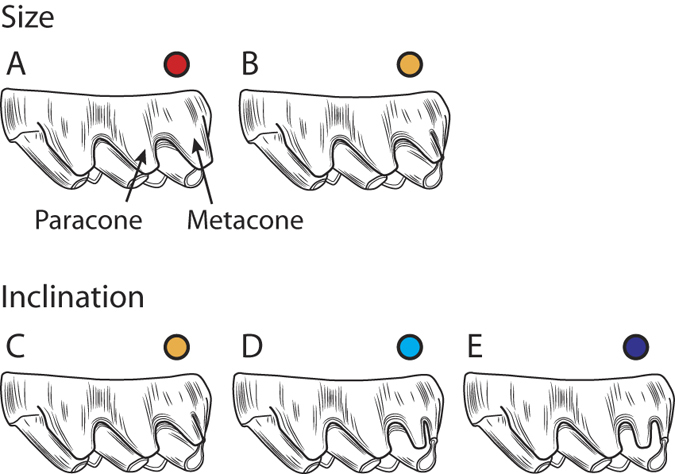
Size and inclination of the metacone on the labial side of the M1 tooth. (**A**) As large as the paracone. (**B**) Smaller than the paracone. (**C**) Inclined posteriorly parallel to the axis of the paracone. (**D**) Slightly inclined posteriorly but not parallel to the axis of the paracone. (**E**) Not inclined posteriorly (=vertical). Colors correspond to those in Fig. 4.

**Figure 3:**
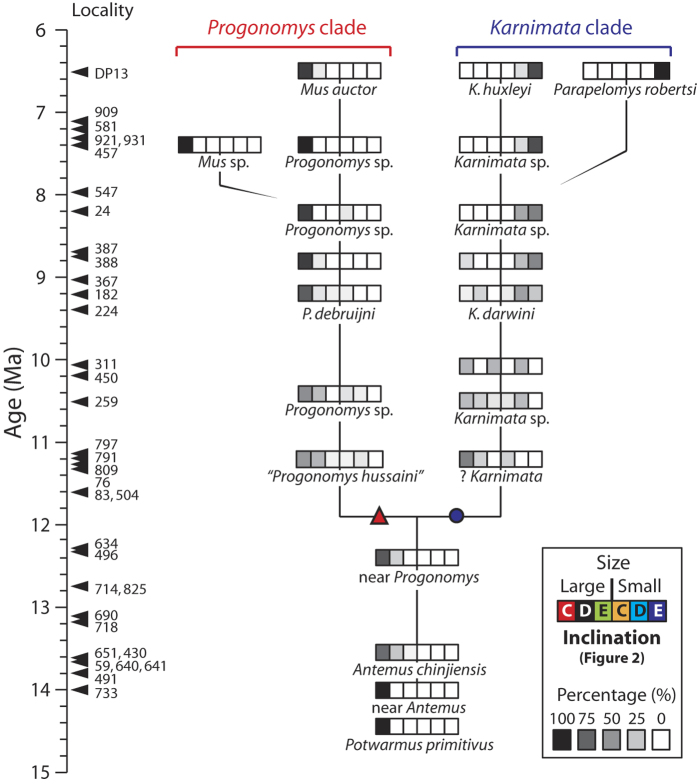
Percent frequency distribution of the size and inclination of the metacone in Siwalik murine rodents. Species that are likely in ancestor-descendant relationships^10^,^33^ are connected by a solid line. Open symbols correspond to those in Fig. 1A, and colors of the ‘size and inclination’ legend correspond to those in Fig. 4. Different from the previous phylogenetic hypothesis (Fig. 1A), *Progonomys hussaini* is interpreted to appear after the initiation of lineage separation^34^. See Tables S1–S3 for numeric percentages and the number of individuals observed in this study, and SI Discussion for age determination. All localities but DP 13 have Y as a prefix.

**Figure 4:**
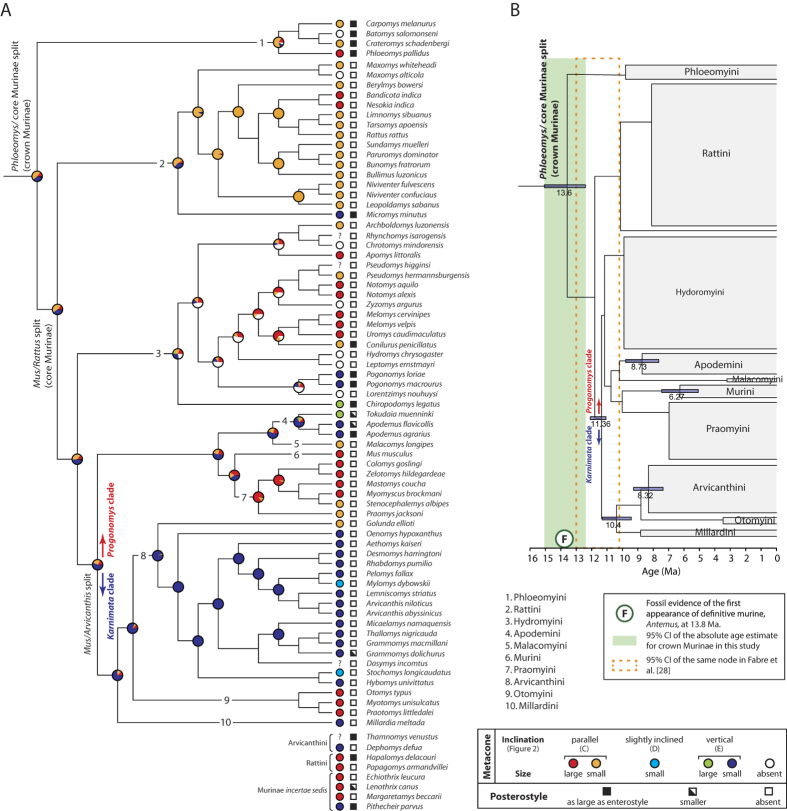
Siwalik murine rodents documenting the *Mus*/*Arvicanthis* split and divergence time estimates. (**A**) Size and inclination of the metacone, as well as the presence of the posterostyle, in modern murine rodents on a cladogram based on the maximum likelihood tree of Fabre *et al.*^28^. Pie charts indicate the probability of ancestral states of the combined characters of the metacone at a given node. Systematic nomenclature of the tribes follows Lecompte *et al.*^22^. The “?” state is assigned for the species in which the inclination of the metacone is not recognizable because the metacone is fused with the paracone or hypocone. (**B**) Maximum clade credibility tree from the BEAST analysis of the Fabre *et al.*^29^ data using the 11.2 Ma calibration point for the *Mus*/*Arvicanthis* split. Node bars indicate the 95% credible interval of the posterior density of divergence times. Number on the nodes represents the posterior mean of divergence times.

First, we examined change in the frequency distribution of the size and inclination of the metacone relative to the paracone on M1 of the Siwalik fossil species. Schematic diagrams of the character states, to which each specimen was referred for scoring characters, are shown in Fig. 2. The size of the metacone was observed as the width of the metacone on the labial side of the tooth relative to that of the paracone, scored as (a) as large as the paracone or (b) smaller than the paracone (Fig. 2A,B). The inclination of the metacone was scored as the axis of the metacone is (c) inclined posteriorly parallel to that of the paracone, (d) slightly inclined posteriorly but not parallel to that of the paracone, and (e) not inclined posteriorly (=vertical) (Fig. 2C–E). The size of the posterostyle was scored relative to that of the enterostyle in occlusal view. These character states were also scored for modern species. We preferred the qualitative assessments, rather than quantitative measurements, in this study because large-scale systematic differences were expected to appear as fixed characters. For the *Karnimata* and *Progonomys* clades, independence and correlation of the two metacone characters were tested and measured by a generalized Cochran-Mantel-Haenszel (CMH) test and Goodman-Kruskal’s gamma.

Then, we traced ancestral states of these metacone characters on the maximum likelihood (ML) tree of Fabre *et al.*^28^ by the ML method of ancestral state reconstruction in Mesquite 2.75^45^. Topologies of molecular trees are largely congruent for Murinae^22^,^24^,^27^,^28^,^30^, but the chronogram of Fabre *et al.*^28^ most comprehensively covers murine species to date. The Markov k-state 1 parameter model (Mk1) was chosen, which takes the rate of character change as a single parameter and assumes equal probability for any particular character change. The likelihood decision threshold of 2.0 was adopted as a cutoff for the significance of the likelihood ratio between two character states. Eight genera (*Thamnomys*, *Dephomys*, *Hapalomys*, *Papagomys*, *Echiothrix*, *Lenothrix*, *Margaretamys*, *Pithecheir*) were excluded from the ancestral character state reconstruction due to low bootstrap values (<70%) on their nodes.

Finally, we applied the new fossil calibration point (the *Mus/Arvicanthis* split) to a published dataset to test whether the new calibration point gives more accurate estimates of absolute nodal ages than previous applications. The data of Fabre *et al.*^29^ were chosen for the test analysis because they provide detailed comparisons of estimated ages obtained by different applications of the 12 Ma fossil date. In our analysis, we replaced these calibration points with the *Mus*/*Arvicanthis* split. The fossil date of the Apodemurini was excluded because its credible interval (CI) greatly overlaps with the newly proposed date for the *Mus*/*Arvicanthis* split. All other conditions were identical to those in Fabre *et al.*^29^. The analysis was conducted in BEAST (v 1.8.0)^46^ via the CIPRES Science Gateway^47^. We bounded the lower and upper limits of this prior (median age: 11.63 Ma, 95% CI: 11.16–14.03 Ma) based on the stratigraphic occurrence of ?*Karnimata* and *Progonomys hussaini* at the lower bound and the stratigraphic occurrence of “near *Progonomys*” at the upper bound, respectively, as described in the Discussion. We set the minimum age of the fossil locality in the 5% quantile of the lognormal curve. SI Materials and Methods provide further details of the methods used in this study.

## Additional Information

**How to cite this article**: Kimura, Y. *et al.* Corrected placement of *Mus-Rattus* fossil calibration forces precision in the molecular tree of rodents. *Sci. Rep.* **5**, 14444; doi: 10.1038/srep14444 (2015).

## Supplementary Material

Supplementary Dataset S1

Supplementary Dataset S2

Supplementary Information
