# Supplementary material for: Corrected placement of Mus-Rattus fossil calibration forces precision in the molecular tree of rodents
Source: Sci Rep. 2015 Sep 28;5:14444. doi: 10.1038/srep14444 (PMC4585935; doi:10.1038/srep14444)
Supplement: Supplementary Information [file srep14444-s3.pdf]

Supplementary Information

Corrected placement of *Mus-Rattus* fossil calibration forces precision in the molecular tree of rodents

Yuri Kimura,
Melissa T. R. Hawkins, Molly M. McDonough, Louis L. Jacobs, Lawrence J. Flynn

Supplementary Information

SI Materials and Methods

(a) Fossil and modern murine rodents

Fossil specimens used in this study were collected from the Potwar Plateau, northern Pakistan, as part of collaborative research between the Geological Survey of Pakistan and several American universities[46,47]. They are on long-term loan from the Geological Survey of Pakistan and are housed in the Peabody Museum of Archaeology and Ethnology, Harvard University. We selected complete M1 in wear stages I to III, which were used in Kimura et al.[34,45] (see Figure S2 of Kimura et al.[34] for wear stage), and also included specimens that are partially broken or slightly weathered so long as they hold complete paracone and metacone. Twelve *Potwarmus primitivus* specimens from YGSP 709 (14.3 Ma) were newly added to the database of the previous studies[34,45]. A total of 272 specimens, ranging from 14.3 to 6.5 Ma, were analyzed in this study (Dataset S1). We follow Kimura et al.[36] for the systematics of Siwalik murines (also see Table S1 of Kimura et al.[45]) although other studies have proposed synonymy of *Progonomys hussaini* with *P. cathalai* and *Karnimata darwini* with *P. woelferi*[48,49]. Minor changes of specimen designation, differing from Kimura et al.[34,45], reflect on-going studies of the Siwalik specimens (Dataset S1). YGSP stands for Yale-Geological Survey of Pakistan, and DP stands for Dartmouth-Peshawar University.

Modern specimens examined in this study (Dataset S2) comprise a total of 500 individuals from 70 genera and 79 species across the murine systematic spectrum, which include at least one genus from each tribe[22] and 25 out of 29 divisions[38]. They cover 54% (70/130) of the total genera of the Murinae and 79% (31/39) of the genera within the *Mus*/*Arvicanthis* split[22,29,38]. These specimens are curated in the Museum of Comparative Zoology (MCZ), Harvard University, and in the Smithsonian Institution National Museum Natural History (USNM). Digital images of upper molars of these species are provided in Figure S3. Permission to photograph these specimens was obtained through MCZ.

(b) Qualitative characters of the metacone

Qualitative characters were preferred in this study because large-scale systematic differences, at the tribal level, were expected to appear as fixed characters. We examine change in the frequency distribution of the size and inclination of the metacone on M1 in Siwalik murine species. Although characters of the anterostyle and enterostyle show lineage separation of

Siwalik murine rodents[33,34,45], we did not utilize these characters in this study because they are, if partly, ecomorphological traits as we previously discussed in Kimura et al.[34].

The frequency distributions of the size and inclination of the metacone, relative to the paracone, were examined for Siwalik murine species. Figure 2 shows schematic diagrams of the character states, to which each specimen was referred for scoring characters. The size of the metacone was observed as the width of the metacone on the labial side of the tooth relative to that of the paracone, scored as (a) as large as the paracone or (b) smaller than the paracone (Figure 2A, B). The inclination of the metacone was scored as the axis of the metacone is (c) inclined posteriorly parallel to that of the paracone, (d) slightly inclined posteriorly but not parallel to that of the paracone, and (e) not inclined posteriorly (=vertical) (Figure 2C-E). The size of the posterostyle was scored relative to that of the enterostyle in occlusal view. The condition that a tooth edge between the enterostyle and hypocone is slightly swollen but not forming a cusp is considered as the absence of the posterostyle. To avoid biased observations leading to false trends, fossil specimens were observed randomly in terms of chronology and systematics. In modern murines, when some variations were recognized within a species, the major variation (>50% occurrence) was chosen as the character state in the species.

For each clade of Siwalik murines, independence and correlation of the two characters of the metacone were tested and measured by a generalized Cochran-Mantel-Haenszel (CMH) test and Goodman-Kruskal's gamma, which take into account ordinality in the variables, in the 'vcd' and 'vcdExtra' packages of R[50,51].

(c) Ancestral state reconstruction in a molecular-based tree

For evolutionary relationships of the Murinae, in which phylogenetic signals can be obscured due to simplicity of the cuspidate teeth, a molecular phylogenetic framework is more robust than dental morphology-based phylogeny. The topologies of molecular trees are largely agreed for the Murinae[22,24,27,28,30]. Most recently, comprehensive molecular phylogenetic trees are reconstructed at the species level, covering 60 to 70% of all murine genera[28,30]. In this study, we utilized the chronogram of Fabre et al.[28], which includes the largest number of murine species to date, in order to reconstruct ancestral states of the metacone characters for the modern murine species. The ancestral states were reconstructed by a maximum likelihood method and were mapped on the ML tree of Fabre et al.[28] using Mesquite 2.75[39]. Among the observed 70 genera, eight genera were excluded from ancestral state reconstruction because their nodal supports are

less than 70% bootstrap values[28]. They are arvicanthine *Thamnomys* and *Dephomys*, rattine *Hapalomys* and *Papagomys*, and four genera (*Echiothrix*, *Lenothrix*, *Margaretamys*, *Pithecheir*) that are assigned to Murinae *incertae sedis*[22,38]. The Markov k-state 1 parameter model (Mk1) was chosen, which takes the rate of character change as a single parameter and assumes equal probability for any particular character change. The likelihood decision threshold of 2.0 was adopted as a cutoff for the significance of the likelihood ratio between two character states.

(d) Comparative divergence dating

In order to test whether a new fossil calibration constraint provides more accurate divergence time estimates for molecular phylogenetics, we calibrated absolute nodal ages using our new fossil calibration for the data of Fabre et al.[29] and compared our result with theirs as well as the fossil record. The data of Fabre et al.[29], a concatenated supermatrix of mitochondrial and nuclear data of 204 murine taxa and outgroups, were chosen for our comparative analysis because Fabre et al.[29] compared different usages of the 12.1 Ma fossil date for molecular divergence estimates. They applied the date to the *Mus*/*Rattus* split or to the *Phloeomys*/core Murinae split, or excluded the fossil date in three separate analyses, which allows us to compare our new calibration point with widely-used previous applications. The program BEAST (v 1.8.0)[40] was run on the XSEDE cluster via the CIPRES Science Gateway[41] on the same alignment and parameters as in Fabre et al.[29], with the only modifications being the fossil calibration points. Fabre et al.[29] used six fossil calibration points throughout the murid radiation in question, and for this analysis, we replaced the 12.1 Ma fossil date (calibration point #1) with the new calibration of the *Mus*/*Arvicanthis* split and removed the stem Apodomurini (calibration point #2).

Fossil calibration point #1 estimated the age of the *Mus*/*Rattus* split or the *Phloeomys*/core Murinae split, which has been the best age estimate to date. The prior of this date (median age: 12.1 Ma, 95% credible interval: 10.01 to 22.9 Ma) ranges most of the evolutionary history (~14 million years) of Murinae. This calibration point was replaced by the *Mus*/*Arvicanthis* split, following the results of our specimen-based study. Fossil calibration point #2 was also removed since the mean date (11 Ma) and 95% credible interval (8.9 to 21.8 Ma) greatly overlap those of the *Mus*/*Arvicanthis* calibration point.

Based on the paleontological evidence presented in our study, the mean age for the *Mus*/*Arvicanthis* split was estimated to be 11.2 Ma, with two geological strata constraining the

dates to 11.1-12.3 Ma (see Discussion for details). For the divergence dating analysis, however, we considered a slightly larger CI to account for any discrepancies in age estimates of the geologic localities. The prior of the fossil date was set as a lognormal distribution curve with the 95% CI of 11.16-14.03 Ma (median 11.63 Ma). Because the Siwalik fossils are associated with specific ages based on the magnetostratigraphic and biostratigraphic frameworks, we set the lower limit of the fossil locality in the 5% quantile of the lognormal curve. The median of this distribution curve is 11.63 Ma, and the 95% quantile is 14.03 Ma. We allowed for a slightly higher upper bound to account for any uncertainty in the age estimates.

Four replicates of the BEAST analysis were conducted. In order to evaluate the effects of the priors, an empty alignment was run. The empty alignment yielded poor ESS values, indicating that the priors were not driving the support and topology of the tree. Tracer v.1.5 was used to evaluate the quality of the run, by checking if ESS values were over 200 and if the four run replicates converged. Two out of four trees were combined as a summary tree in Tree Annotator.

SI Discussion

(a) Paleontological hypothesis for early murines

Jacobs[33] and Jacobs and Downs[10] proposed dichotomous lineages deriving from *Antemus* (Figure 1A) as a simplified phylogenetic hypothesis for Siwalik murine rodents. In the Siwalik lineages, the *Progonomys* clade contains taxa with the anterostyle located posteriorly, whereas the *Karnimata* clade is composed of taxa with the anterostyle displaced more anteriorly on M1. *Mus* was placed in the *Progonomys* clade based on observation of gradual morphological change from older *Progonomys* to younger *Mus auctor* in the Siwalik record[10,33,34]. On the other hand, *Rattus* was placed in the *Karnimata* clade with some uncertainty[10], along with extant genera of the Arvicanthini (*Arvicanthis*, *Pelomys*, *Mylomys*, and *Golunda*)[33], heavily relying on the anterior displacement of the anterostyle and an inevitable assumption that Siwalik fossils record essential features of early evolution of the Murinae. Jacobs pointed out that *Golunda*, *Pelomys*, *Mylomys* are close modern relatives to Siwalik *Parapelomys robertsi*, which was considered to be derived from an earlier species of *Karnimata*, based on the crescentic appearance of major cusps and weak connections between the major cusps[33]. However, except for the position of the anterostyle,

no further morphological evidence is found for the evolutionary relationship between *Rattus* and Siwalik species of the *Karnimata* clade[35].

(b) The metacone in the Arvicanthini-Otomyini-Millardini clade

In the Arvicanthini, a small metacone in vertical orientation is present in 12 of 17 genera (Figures 4, S3). Among the remaining five genera of the Arvicanthini, the metacones of the three genera (*Golunda*, *Mylomys*, *Stochomys*) are clearly not vertical. Molecular phylogeny of African murines places *Golunda* in the most basal branch of the tribe and *Mylomys* sister to *Pelomys* in a more derived branch[22,28], which agrees with comparative observations of craniodental characters in the three genera[52]. Nevertheless, derived conditions on lower third molars (m3) shared between *Golunda* and *Mylomys* were pointed out[33]. Differing from the basal murine form, the paracone and metacone are vestigial or absent, the protocone is dominantly large, and the hypocone is connected to the posterior part of the protocone in the two genera (Figure S3). This combination of characters is not present in any other genera observed in this study. The main cusps of *Golunda* and *Mylomys* are inclined more posteriorly than those of other arvicathines, which results in more crescent-shaped cusps. In relation to the strong posterior inclination of the cusps, the occlusal surface of the enterostyle is more strongly crescentic and is larger than that of the paracone, unlike the symmetrical occlusal shape of the two cusps in other arvicanthines (Figure S3). Thus, these two genera probably acquired the posteriorly inclined metacone secondarily, corresponding to the strong posterior inclination of other cusps. *Stochomys* also show a slight inclination of the metacone on M1 although it is vertical on M2. In general, cusps are more lophate (ridge-like) in *Stochomys* than in other arvicanthines (Figure S3). A slight inclination of metacone in *Stochomys* is probably associated with the lophate pattern of molars for increased mastication efficiency.

In the Otomyini, a large metacone is inclined posteriorly parallel to the paracone (Figure S3). *Euryotomys*, a fossil otomyine showing transitional dental morphology from a basal murine plan to laminated teeth, documents a parallel metacone in the early Pliocene[53–56], but the inclination of the metacone in *Euryotomys* is not as strong as that in *Otomys*[54]. Thus, the Otomyini probably developed posterior inclination of the metacone secondarily in their specialized teeth.

(c) The metacone outside the Arvicanthini-Otomyini-Millardini clade

Outside the Arvicanthini-Otomyini-Millardini clade, a vertical metacone has evolved independently in six genera of three tribes. They are *Micromys* in the most basal branch of the Rattini, *Pogonomys* in the Hydromyini, *Chiropodomys* as a sister taxon of the Hydoromyini, both genera (*Tokudaia* and *Apodemus*) in the Apodemini, and *Pithecheir* in Murinae *incertae sedis*. Phylogenetic positions of these genera except *Pithecheir* are resolved by molecular phylogeny with strong statistical support[22,27,28]. Interestingly, all these genera except *Tokudaia* have a well-developed posterostyle that is as large as the enterostyle (hereafter the well-developed posterostyle, Figure S3). The well-developed posterostyle is as vertical as the metacone and is located in a symmetrical position to that cusp. The vertical metacone in these genera may have evolved to correspond to the well-developed posterostyle in vertical orientation. In the Apodemini, the size of the posterostyle varies from smaller than the enterostyle (*Tokudaia muenninki*, *Apodemus flavicollis*) to as large as the enterostyle (*A. agrarius*), but the posterostyle is always present in 36 individuals of the three species examined here. On the other hand, in the Arvicanthini-Otomyini-Millardini clade, the posterostyle is present only in arvicanthine *Grammomys* and *Thamnomys* (2 out of 23 genera, Figure S3). Similarly, none of the individuals of Siwalik murines have the posterostyle.

(d) Magnetostratigraphy and locality ages

One of the clear and compelling features of the geomagnetic time scale is that correctly correlated magnetochrons provide a precise global chronology. The numerical values of the Neogene Geomagnetic Polarity Time Scale were modeled by applying a cubic spline fit to the sea-floor magnetic anomaly pattern calibrated by radiometric age determinations. This method provided specific ages for each chron derived from the resulting magnetic anomaly distance scale (sea floor spreading) with uncertainties expressed in kilometers[57–59]. Refinement has occurred through application of additional radiometric age determinations (e.g., ref [60]) and through astronomical calibration[61]. The time scales of Gradstein et al.[37] and Gradstein et al.[59] are identical in the interval between chrons C5Bn.1n and C4An (14.9-8.8 Ma) with very minor discrepancies in two internal boundaries, 0.065 m.y. (C5An.2n; 12.272 Ma) and -0.031 m.y. (C5ADn; 14.163 Ma). Any other differences between the two time scales are smaller and therefore not significant.

References

1. Ho, S. Y. W. & Phillips, M. J. Accounting for calibration uncertainty in phylogenetic estimation of evolutionary divergence times. *Syst. Biol.* **58,** 367–80 (2009).
2. Ho, S. Y. W. & Duchêne, S. Molecular-clock methods for estimating evolutionary rates and timescales. *Mol. Ecol.* **23,** 5747–5965 (2014).
3. Graur, D. & Martin, W. Reading the entrails of chickens: molecular timescales of evolution and the illusion of precision. *Trends Genet.* **20,** 80–86 (2004).
4. Donoghue, P. C. J. & Benton, M. J. Rocks and clocks: calibrating the Tree of Life using fossils and molecules. *Trends Ecol. Evol.* **22,** 424–431 (2007).
5. Warnock, R. Calibration uncertainty in molecular dating analyses: there is no substitute for the prior evaluation of time priors. *Proc. R. Soc. London B* **282,** 20141013 (2015).
6. Dos Reis, M. & Yang, Z. The unbearable uncertainty of Bayesian divergence time estimation. *J. Syst. Evol.* **51,** 30–43 (2013).
7. Benton, M. J. & Donoghue, P. C. J. Paleontological evidence to date the tree of life. *Mol. Biol. Evol.* **24,** 26–53 (2007).
8. Benton, M. J., Donoghue, P. C. J. & Asher, R. J. in *The Timetree of Life* (eds. Hedges, S. B. & Kumar, S.) 35–86 (Oxford University Press, 2009).
9. Steppan, S., Adkins, R. M. & Anderson, J. Phylogeny and divergence-date estimates of rapid radiations in muroid rodents based on multiple nuclear genes. *Syst. Biol.* **53,** 533–553 (2004).
10. Jacobs, L. L. & Downs, W. R. in *Rodent and Lagomorph Families of Asian Origins and Diversification* (eds. Tomida, Y., Li, C. & Setoguchi, T.) 149–156 (National Science Museum Monographs 8, 1994).
11. Jacobs, L. L. & Flynn, L. J. in *Interpreting the Past: Essays on Human, Primate, and Mammal Evolution in Honor of David Pilbeam* (eds. Lieberman, D. E., Smith, R. J. & Keller, J.) 63–80 (Brill Academic Publishers, 2005).
12. Jacobs, L. L. & Pilbeam, D. Of mice and men: Fossil-based divergence dates and molecular 'clocks'. *J. Hum. Evol.* **9,** 551–555 (1980).
13. She, J. X., Bonhomme, F., Boursot, P., Thaler, L. & Catzeflis, F. Molecular phylogenies in the genus *Mus*: Comparative analysis of electrophoretic, scnDNA hybridization, and mtDNA RFLP data. *Biol. J. Linn. Soc.* **41,** 83–103 (1990).

14. Chevret, P., Denys, C., Jaeger, J.-J., Michaux, J. & Catzeflis, F. Molecular and palaeontological aspects of the tempo and mode of evolution in *Otomys* (Otomyinae: Muridae: Mammalia). *Biochem. Syst. Ecol.* **21,** 123–131 (1993).
15. Chevret, P., Veyrunes, F. & Britton-Davidian, J. Molecular phylogeny of the genus *Mus* (Rodentia: Murinae) based on mitochondrial and nuclear data. *Biol. J. Linn. Soc.* **84,** 417–427 (2005).
16. Smith, M. F. & Patton, J. L. Phylogenetic relationships and the radiation of sigmodontine rodents in South America: evidence from cytochrome b. *J. Mamm. Evol.* **6,** 89–128 (1999).
17. Ducroz, J. F., Volobouev, V. & Granjon, L. An assessment of the systematics of arvicanthine rodents using mitochondrial DNA sequences: evolutionary and biogeographical implications. *J. Mamm. Evol.* **8,** 173–206 (2001).
18. Michaux, J., Reyes, A. & Catzeflis, F. Evolutionary history of the most speciose mammals: molecular phylogeny of muroid rodents. *Mol. Biol. Evol.* **18,** 2017–2031 (2001).
19. Salazar-Bravo, J., Dragoo, J. W., Tinnin, D. S. & Yates, T. L. Phylogeny and evolution of the neotropical rodent genus *Calomys*: inferences from mitochondrial DNA sequence data. *Mol. Phylogenet. Evol.* **20,** 173–184 (2001).
20. Huchon, D. *et al.* Rodent phylogeny and a timescale for the evolution of Glires: evidence from an extensive taxon sampling using three nuclear genes. *Mol. Biol. Evol.* **19,** 1053–1065 (2002).
21. Suzuki, H., Shimada, T., Terashima, M., Tsuchiya, K. & Aplin, K. Temporal, spatial, and ecological modes of evolution of Eurasian *Mus* based on mitochondrial and nuclear gene sequences. *Mol. Phylogenet. Evol.* **33,** 626–646 (2004).
22. Lecompte, E. *et al.* Phylogeny and biogeography of African Murinae based on mitochondorial and nuclear gene sequences, with a new tribal classification of the subfamily. *BMC Evol. Biol.* **8,** 199 (2008).
23. Watts, C. & Baverstock, P. Evolution in the Murinae (Rodentia) assessed by microcomplement fxation of albumin. *Aust. J. Zool.* **43,** 105–118 (1995).
24. Steppan, S. J., Adkins, R. M., Spinks, P. Q. & Hale, C. Multigene phylogeny of the Old World mice, Murinae, reveals distinct geographic lineages and the declining utility of mitochondrial genes compared to nuclear genes. *Mol. Phylogenet. Evol.* **37,** 370–388 (2005).
25. Jansa, S. A. & Weksler, M. Phylogeny of muroid rodents: relationships within and among major lineages as determined by IRBP gene sequences. *Mol. Phylogenet. Evol.* **31,** 256–276 (2004).

26. Jansa, S. A., Barker, F. K. & Heaney, L. R. The pattern and timing of diversification of Philippine endemic rodents: evidence from mitochondrial and nuclear gene sequences. *Syst. Biol.* **55,** 73–88 (2006).

27. Rowe, K. C., Reno, M. L., Richmond, D. M., Adkins, R. M. & Steppan, S. J. Pliocene colonization and adaptive radiations in Australia and New Guinea (Sahul): multilocus systematics of the old endemic rodents (Muroidea: Murinae). *Mol. Phylogenet. Evol.* **47,** 84–101 (2008).

28. Fabre, P.-H., Hautier, L., Dimitrov, D. & Douzery, E. J. P. A glimpse on the pattern of rodent diversification: a phylogenetic approach. *BMC Evol. Biol.* **12,** 88 (2012).

29. Fabre, P. *et al.* A new genus of rodent from Wallacea (Rodentia: Muridae: Murinae: Rattini), and its implication for biogeography and Indo-Pacific Rattini systematics. *Zool. J. Linn. Soc.* **169,** 408–447 (2013).

30. Schenk, J. J., Rowe, K. C. & Steppan, S. J. Ecological opportunity and incumbency in the diversification of repeated continental colonizations by muroid rodents. *Syst. Biol.* **62,** 837–64 (2013).

31. Rowe, K. C., Aplin, K. P., Baverstock, P. R. & Moritz, C. Recent and rapid speciation with limited morphological disparity in the genus Rattus. *Syst. Biol.* **60,** 188–203 (2011).

32. Musser, G. G. & Heaney, L. Philippine rodents: definitions of *Tarsomys* and *Limnomys* plus a preliminary assessment of phylogenetic patterns among native Philippine murines (Murinae, Muridae). *Bull. Am. Museum Nat. Hist.* **211,** 1–138 (1992).

33. Jacobs, L. L. Fossil rodents (Rhizomyidae and Muridae) from Neogene Siwalik deposits, Pakistan. *Museum North. Arizona Press Bull. Ser.* **52,** 1–103 (1978).

34. Kimura, Y., Jacobs, L. L. & Flynn, L. J. Lineage-specific responses of tooth shape in murine rodents (Murinae, Rodentia) to late Miocene dietary change in the Siwaliks of Pakistan. *PLoS One* **8,** e76070 (2013).

35. Patnaik, R. Phylogeny of Siwalik murine rodents: Implications for *Mus-Rattus* divergence time. *J. Paleontol. Soc. India* **59,** 15–28 (2014).

36. Kimura, Y., Flynn, L. J. & Jacobs, L. L. A paleontological case study for species delimitation in diverging fossil lineages. *Hist. Biol.* (2015).

37. Gradstein, F. M., Ogg, J. G. & Grastein, F. M. Geologic Time Scale 2004 - why, how, and where next! *Lethaia* **37,** 175–181 (2004).

38. Musser, G. G. & Carleton, M. D. in *Mammal species of the World: A taxonomic and Geographic Reference* (eds. Wilson, D. E. & Reeder, D. M.) 894–1531 (Johns Hopkins University Press, 2005).

39. Maddison, W. P. & Maddison, D. R. Mesquite: a modular system for evolutionary analysis. version 2.75. (2011).
40. Drummond, A. J., Suchard, M. A., Xie, D. & Rambaut, A. Bayesian phylogenetics with BEAUti and the BEAST 1.7. *Mol. Biol. Evol.* **29,** 1969–1973 (2012).
41. Miller, M. A., Pfeiffer, W. & Schwartz, T. Creating the CIPRES science gateway for inference of large phylogenetic trees. *Proc. Gatew. Comput. Environ. Work. (GCE), New Orleans, LA* **14,** 1 – 8 (2010).
42. Martín-Suárez, E. & Mein, P. Revision of the genera *Parapodemus*, *Apodemus*, *Rhagamys* and *Rhagapodemus* (Rodentia, Mammalia). *Geobios* **31,** 87–97 (1998).
43. Parham, J. F. *et al.* Best practices for justifying fossil calibrations. *Syst. Biol.* **61,** 346–359 (2012).
44. Ogg, J. G. & Smith, A. G. in *A Geologic Time Scale 2004* (eds. Gradstein, F. M., Ogg, J. G. & Smith, A. G.) 63–86 (Cambridge University Press, 2004).
45. Kimura, Y. *et al.* Fossil mice and rats show isotopic evidence of niche partitioning and change in dental ecomorphology related to dietary shift in Late Miocene of Pakistan. *PLoS One* **8,** e69308 (2013).
46. Barry, J. C., Lindsay, E. H. & Jacobs, L. L. A biostratigraphic zonation of the middle and upper Siwaliks of the Potwar Plateau of northern Pakistan. *Palaeogeogr. Palaeoclimatol. Palaeoecol.* **37,** 95–130 (1982).
47. Barry, J. C. *et al.* Faunal and environmental change in the late Miocene Siwaliks of northern Pakistan. *Paleobiol. Mem.* **3,** 1–71 (2002).
48. Mein, P., Martín Suárez, E. & Agustí, J. *Progonomys* Schaub, 1938 and *Huerzelerimys* gen. nov. (Rodentia); their evolution in Western Europe. *Scr. Geol.* **103,** 41–63 (1993).
49. Wessels, W. Miocene rodent evolution and migration: Muroidea from Pakistan, Turkey and North Africa. *Geol. Ultraiectina* **307,** 1–290 (2009).
50. Meyer, D., Zeileis, A. & Hornik, K. vcd: Visualizing Categorical Data. *R Package version 1.3-2*. (2014).
51. Friendly, M. vcdExtra: vcd extensions and additions. *R Package version 0.6-1*. (2014).
52. Musser, G. G. The occurrence of Hadromys (Rodentia: Muridae) in early Pleistocene Siwalik strata in northern Pakistan and its bearing on biogeographic affinities between India and northeastern African murine faunas. *Am. Museum Novit.* **2883,** 1–36 (1987).
53. Pocock, T. N. Pliocene mammalian microfauna from Langebaanweg: a new fossil genus linking the Otomyinae with the Murinae. *S. Afr. J. Sci.* **72,** 58–60 (1976).

54. Denys, C., Michaux, J. & Hendey, B. Les rongeurs (Mammalia) Euryotomys et *Otomys*: un exemple d'évolution parallèle en Afrique tropicale? *Comptes rendus l'Académie des Sci. Série 2, Mécanique, Phys. Chim. Sci. l'univers, Sci. la Terre* **305,** 1389–1395 (1987).
55. Sénégas, F. & Avery, D. M. New evidence for the murine origins of the Otomyinae (Mammalia, Rodentia) and the age of Bolt's Farm (South Africa). *S. Afr. J. Sci.* **94,** 503–507 (1998).
56. Sénégas, F. in *African Small Mammals* (eds. Denys, C., Granjon, L. & Poulet, A.) 151–160 (IRD Éditions, Collection Colloques et Séminaires, 2001).
57. Cande, S. C. & Kent, D. V. A new geomagnetic polarity time scale for the Late Cretaceous and Cenozoic. *J. Geophys. Res.* **97,** 917–951 (1992).
58. Cande, S. C. & Kent, D. V. Revised calibration of the geomagnetic polarity timescale for the Late Cretaceous and Cenozoic. *J. Geophys. Res.* **100,** 6093–6095 (1995).
59. Gradstein, F. M., Ogg, J. G., Schmitz, M. & Ogg, G. The Geologic Time Scale 2012. (Elsevier, 2012)
60. Berggren, W. A. *et al.* Late Neogene chronology: New perspectives in high-resolution stratigraphy. *Geol. Soc. Am. Bull.* **107,** 1272–1287 (1995).
61. Hilgen, F. J., Lourens, L. J. & Van Dam, J. A. in *A Geological Time Scale 2012* (eds. Gradstein, F. M., Ogg, J. G., Schmitz, M. D. & Ogg, G.) 923–978. (Elsevier, 2012).

Table S1. Percent frequency distribution of the size and inclination of the metacone in early murine rodents from the Siwalik Group, corresponding to Figure 3. The number of specimens is shown in parentheses.

Age (Ma)	Species	N	Large metacone			Small metacone			Combined age
			Inclination of metacone			Inclination of metacone			
			Inclined, Parallel	Inclined, Not parallel	Vertical	Inclined, Parallel	Inclined, Not parallel	Vertical	
12.3	near *Progonomys*	5	80% (4)	20% (1)	0% (0)	0% (0)	0% (0)	0% (0)	12.4, 12.3 Ma
13.6	*Antemus chinjiensis*	24	71% (17)	25% (6)	4% (1)	0% (0)	0% (0)	0% (0)	13.8, 13.7, 13.6, 13.2, 13.1, 12.8 Ma
14.1	near *Antemus*	4	100% (4)	0% (0)	0% (0)	0% (0)	0% (0)	0% (0)	
14.3	*Potwarmus primitivus*	12	100% (12)	0% (0)	0% (0)	0% (0)	0% (0)	0% (0)	

Table S2. Percent frequency distribution of the size and inclination of the metacone in the *Karnimata* clade, corresponding to Figure 3. The number of specimens is shown in parentheses.

Age (Ma)	Species	N	Large metacone			Small metacone			Combined age
			Inclination of metacone			Inclination of metacone			
			Inclined, Parallel	Inclined, Not parallel	Vertical	Inclined, Parallel	Inclined, Not parallel	Vertical	
6.5	*Parapelomys robertsi*	4	0% (0)	0% (0)	0% (0)	0% (0)	0% (0)	100% (4)	
6.5	*Karnimata huxleyi*	7	0% (0)	0% (0)	0% (0)	0% (0)	14% (1)	86% (6)	
7.4	*Karnimata* sp.	13	0% (0)	0% (0)	0% (0)	0% (0)	15% (2)	85% (11)	
8.2	*Karnimata* sp.	15	0% (0)	0% (0)	0% (0)	0% (0)	40% (6)	60% (9)	
8.8	*Karnimata* sp.	7	14% (1)	0% (0)	0% (0)	0% (0)	29% (2)	57% (4)	
9.2	*Karnimata darwini*	37	5% (2)	19% (7)	0% (0)	8% (3)	43% (16)	24% (9)	9.4, 9.2, 9.0 Ma
10.1	*Karnimata* sp.	6	33% (2)	0% (0)	33% (2)	0% (0)	33% (2)	0% (0)	
10.5	*Karnimata* sp.	10	30% (3)	20% (2)	10 (1)	10% (1)	30% (3)	0% (0)	10.5, 10.2 Ma
11.2	? *Karnimata*	5	60% (3)	20% (1)	0% (0)	20% (1)	0% (0)	0% (0)	

Table S3. Percent frequency distribution of the size and inclination of the metacone in the *Progonomys* clade, corresponding to Figure 3. The number of specimens is shown in parentheses.

Age (Ma)	Species	N	Large metacone			Small metacone			Combined age
			Inclination of metacone			Inclination of metacone			
			Inclined, Parallel	Inclined, Not parallel	Vertical	Inclined, Parallel	Inclined, Not parallel	Vertical	
6.5	*Mus auctor*	12	92% (11)	8% (1)	0% (0)	0% (0)	0% (0)	0% (0)	
7.4	*Mus* sp.	8	100% (8)	0% (0)	0% (0)	0% (0)	0% (0)	0% (0)	
7.4	*Progonomys* sp.	7	100% (7)	0% (0)	0% (0)	0% (0)	0% (0)	0% (0)	
8.2	*Progonomys* sp.	11	91% (10)	0% (0)	0% (0)	9% (1)	0% (0)	0% (0)	
8.8	*Progonomys* sp.	12	92% (11)	8% (1)	0% (0)	0% (0)	0% (0)	0% (0)	
9.2	*Progonomys debruijni*	18	78% (14)	11% (2)	5.5% (1)	5.5% (1)	0% (0)	0% (0)	9.2, 9.0 Ma
10.5	*Progonomys* sp.	17	53% (9)	29% (5)	0% (0)	12% (2)	6% (1)	0% (0)	10.5, 10.1 Ma
11.3	*Progonomys hussaini*	21	48% (10)	33% (7)	5% (1)	5% (1)	10% (2)	0% (0)	11.6, 11.5, 11.4, 11.3, 11.2 MA

Table S4. Summary of comparative divergence estimates of this study and three separate analyses of Fabre et al.[29]. LowerCI stands for the lower limit of the 95% credible interval. Note that the crown Murinae is mistakenly listed as core Murinae in Fabre et al. [29].

	This study	Fabre's method 1	Fabre's method 2	Fabre's method 3
MRCA (median fossil age)	Mean (LowerCI–UpperCI)	Mean (LowerCI–UpperCI)	Mean (LowerCI–UpperCI)	Mean (LowerCI–UpperCI)
Crown Murinae (13.8 Ma)	13.59 (12.40-15.08)	11.6 (10.2–13.0)	12.1 (10.9–13.6)	11.8 (10.4–13.7)
Core Murinae	11.81 (11.11-12.68)	10.1 (9.2–11.3)	10.5 (9.7–11.6)	10.3 (9.2–11.6)
Mus/Arvicanthis split (11.2 Ma)	11.36 (11.07-12.1)	9.6 (8.8-10.8)	9.9 (9.2-11.1)	9.6 (8.8-10.8)
Arvicanthini-Otomyini-Millardini (9.2 Ma)	10.4 (9.43-11.33)	8.8 (7.9-10.0)	9.1 (8.2-10.3)	8.9 (7.9-10.0)
Murini (7.4 Ma)	6.27 (5.08-7.46)	5.5 (4.6–6.6)	5.6 (4.7–6.6)	5.6 (4.7–6.6)
Apodemini (8.7-9.7 Ma)	8.73 (7.65-9.80)	7.6 (6.8-8.6)	7.8 (6.9-8.8)	7.6 (6.8-8.9)
Nodal assignment	*Mus/Arvicanthis*	*Phloeomys*/core Murinae	*Mus/Rattus*	Not used
Paleontological event used	Initiation of lineage separation	First appearance of *Progonomys*	First appearance of *Progonomys*	Not applicable
Prior age (LowerCI to UpperCI)	11.11 to 12.68	10.01 to 22.9	10.01 to 22.9	Not applicable

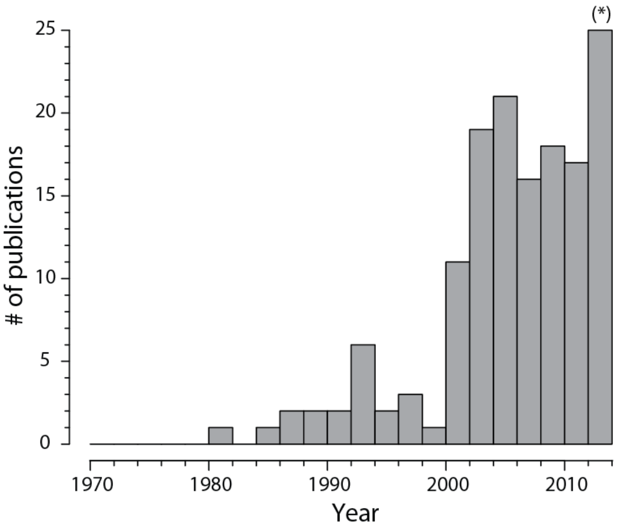

Figure S1. Google Scholar search result for scientific articles that contain discussion about Siwalik murine fossils as a fossil calibration point for divergence time estimates. An asterisk indicates that the number of articles in 2014 is included in the time bin of 2012 to 2014. Searched keywords are listed in Dataset S1.

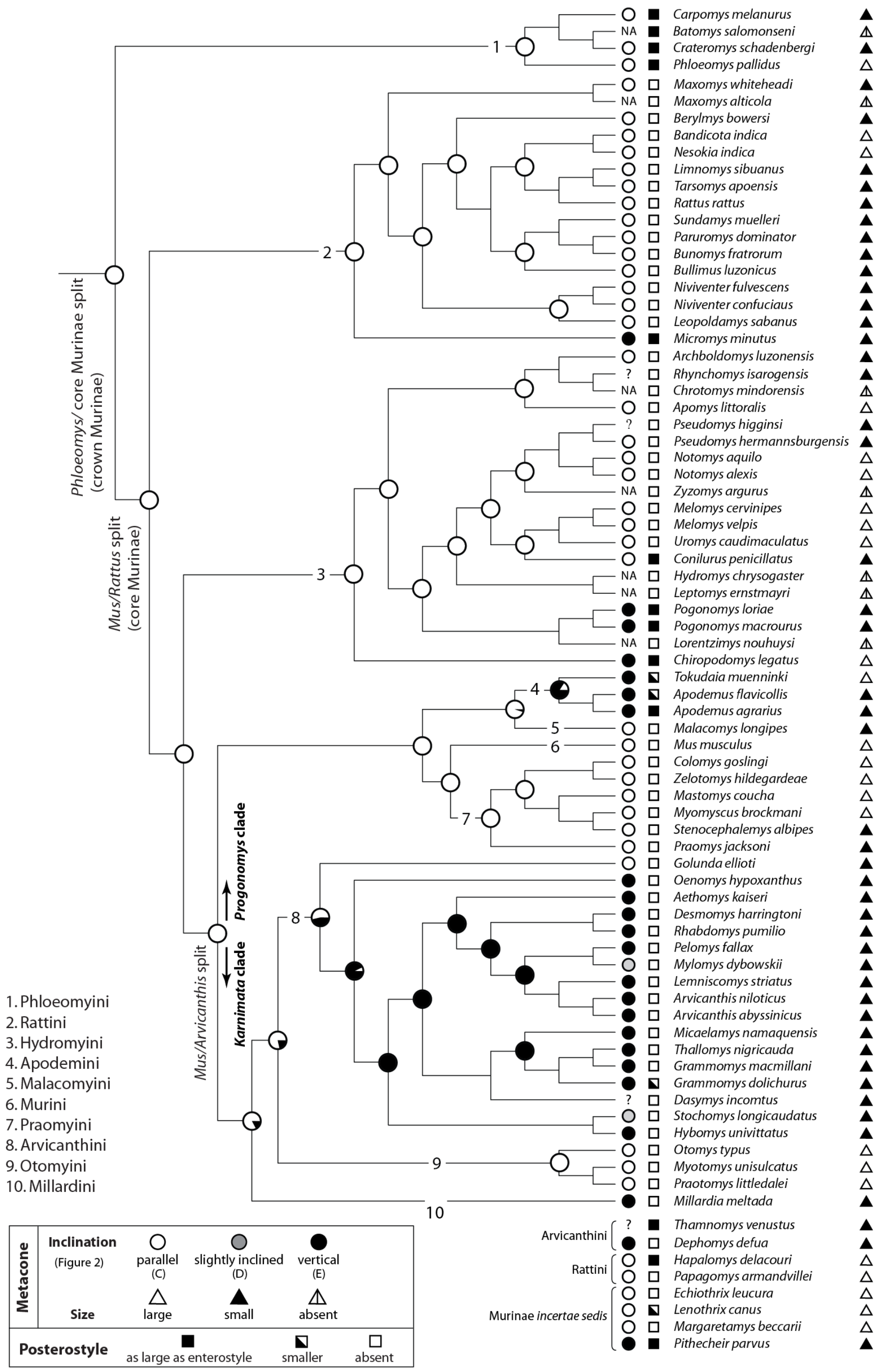

Figure S2. Size and inclination of the metacone, as well as the presence of a well-developed posterostyle, in modern murine rodents on a cladogram based on the maximum likelihood tree of Fabre et al.[28]. Pie charts indicate the probability of ancestral states of the inclination of the metacone at a given node. Systematic nomenclature of the tribes follows Lecompte et al.[22].

Phloeomyini

Crateromys schadenbergi, MCZ 35040

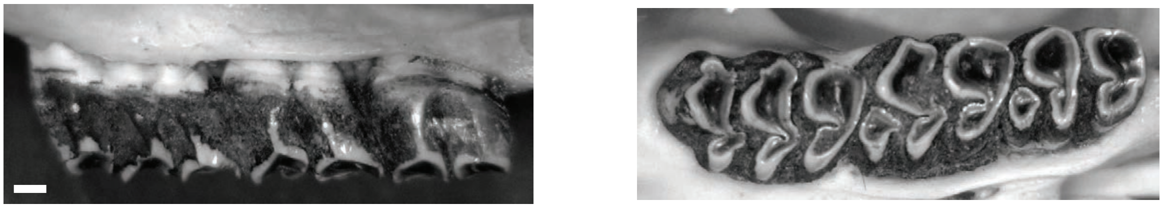

Phloeomys pallidus, MCZ 35041

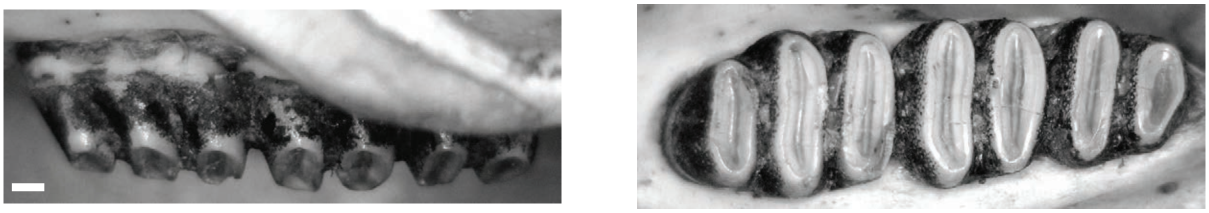

Rattini

Maxomys alticola, USNM 292840

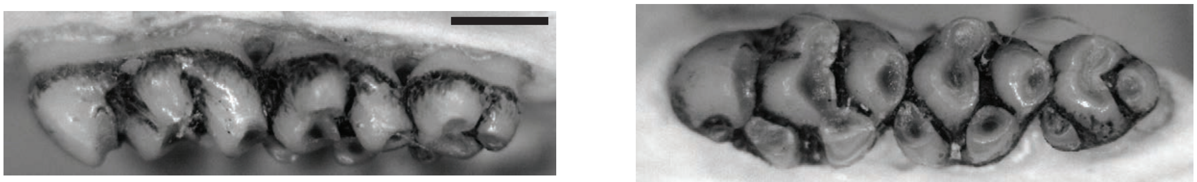

Berylmys bowersi, MCZ 24512

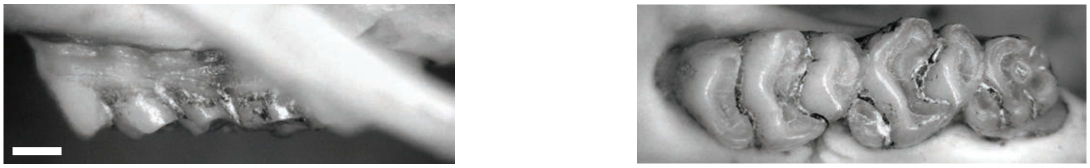

Bandicota indica, MCZ 29473

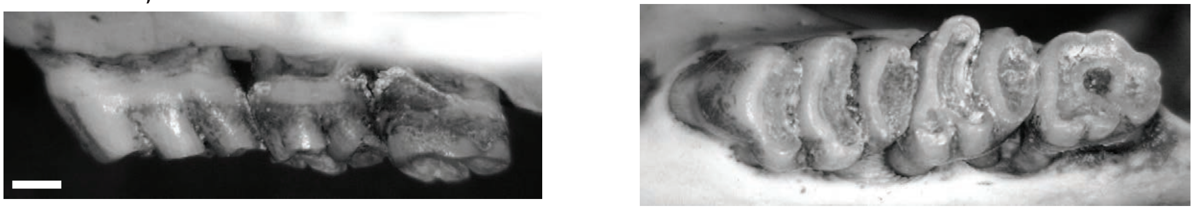

Nesokia indica, MCZ 25329

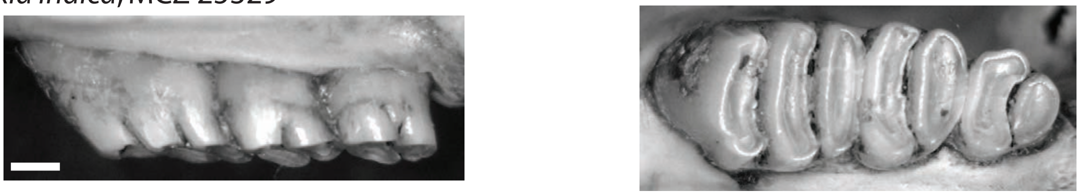

Rattus rattus, MCZ 38873

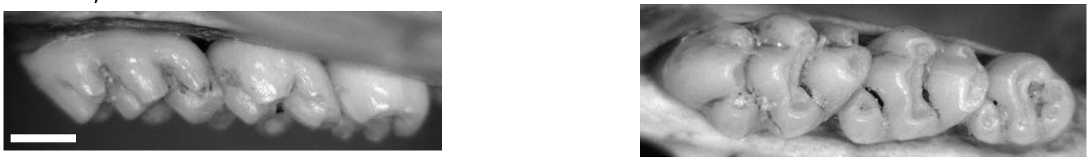

Figure S3. Upper first molars of selected modern murine rodents analyzed in this study. Photographs show the labial side (left) and the occlusal surface (right). Scale bars equal 1 mm. The labial side and occlusal surface of the same specimen are at the same scale.

Rattini (*Continued*)

Sundamys muelleri, MCZ 8724

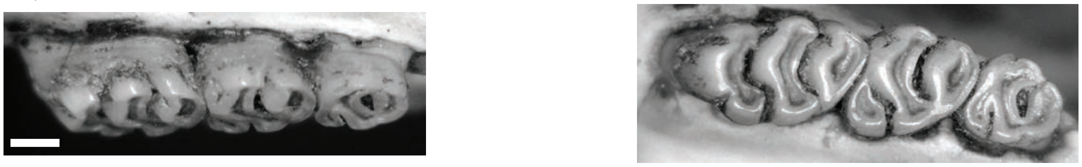

Bunomys fratrorum, USNM 217612

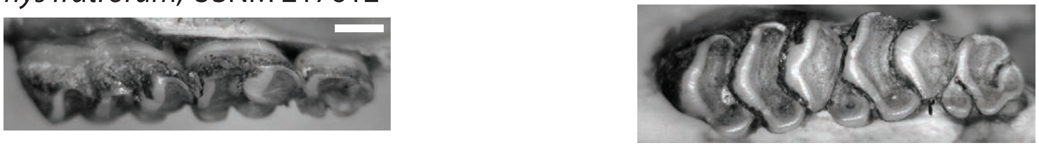

Bullimus luzonicus, USNM 151505

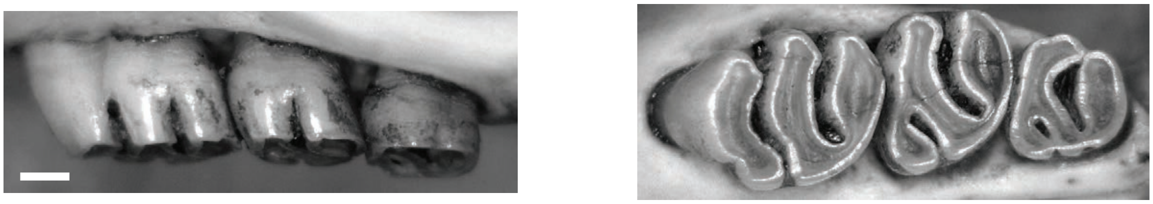

Niviventer confucianus, MCZ 24272

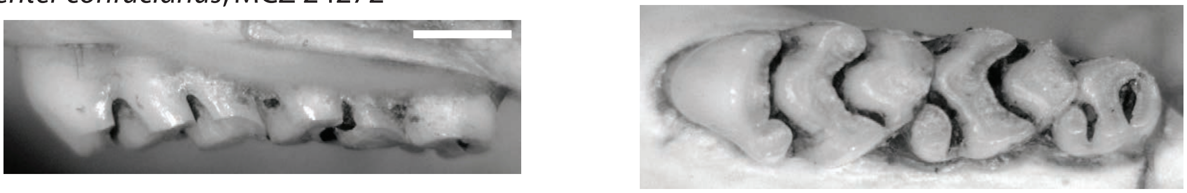

Leopoldamys sabanus, MCZ 36921

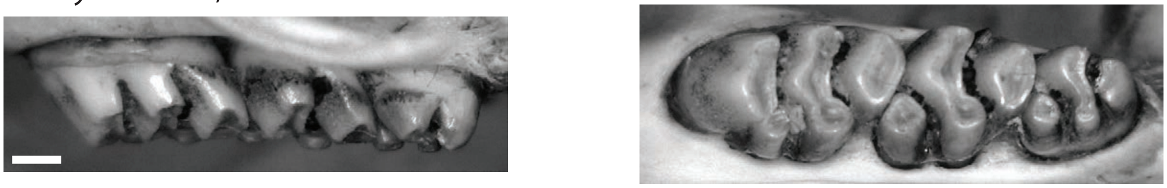

Micromys minutus, MCZ 43403

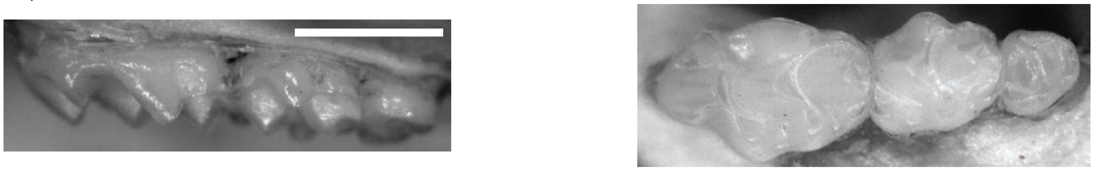

Hydromyini

Rhynchomys isarogensis, USNM 573576

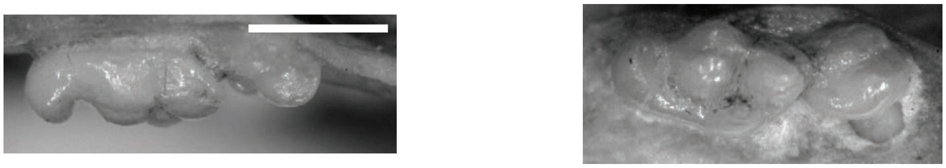

Chrotomys mindorensis, USNM 536801

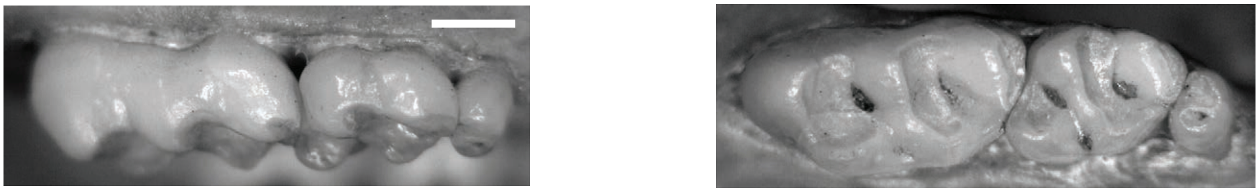

Hydromyini (*Continued*)

Apomys littoralis, USNM 458763

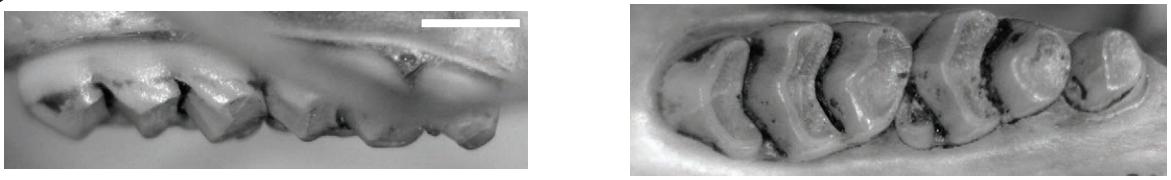

Pseudomys hermannsburgensis, MCZ 29406

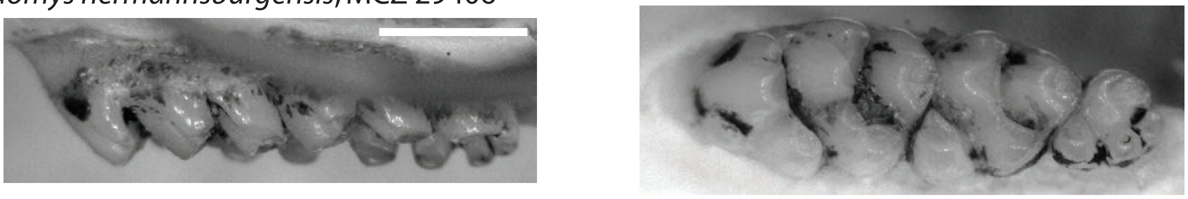

Hydromys chrysogaster, MCZ 29218

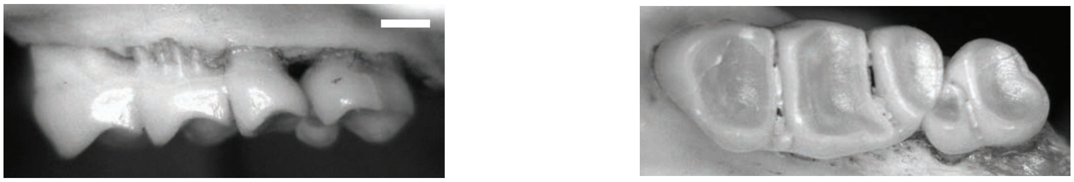

Notomys aquilo, USNM 284355

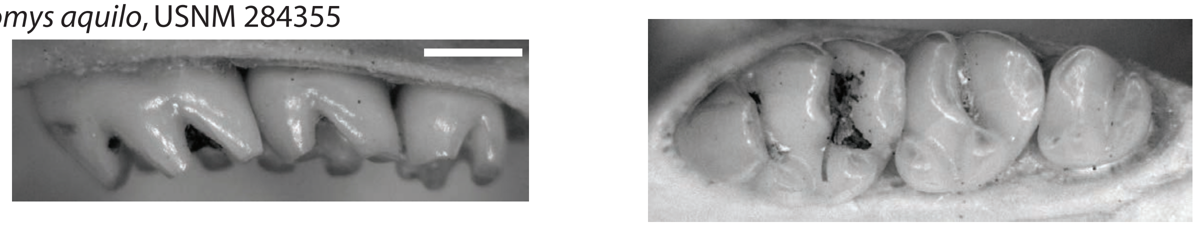

Melomys cervinipes, MCZ 29241

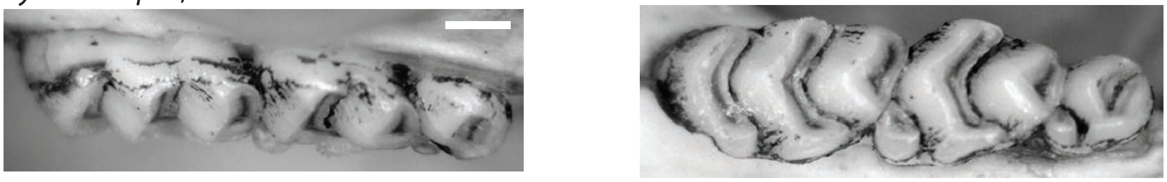

Chiropodomys major, MCZ36535

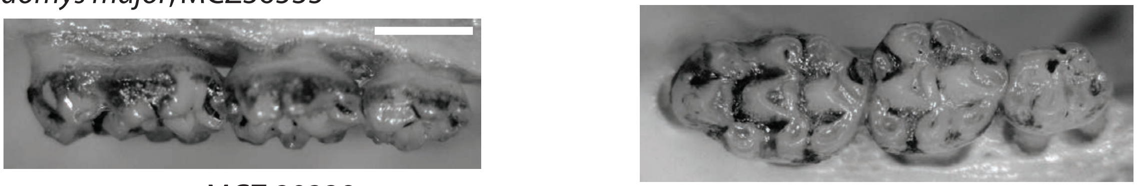

Pogonomys macrourus, MCZ 29338

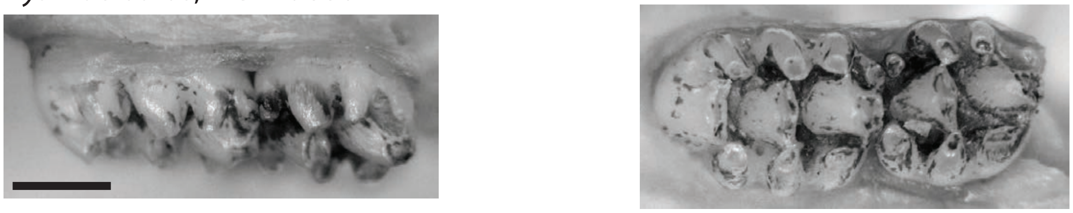

Malacomyini

Malacomys longipes, MCZ 17648

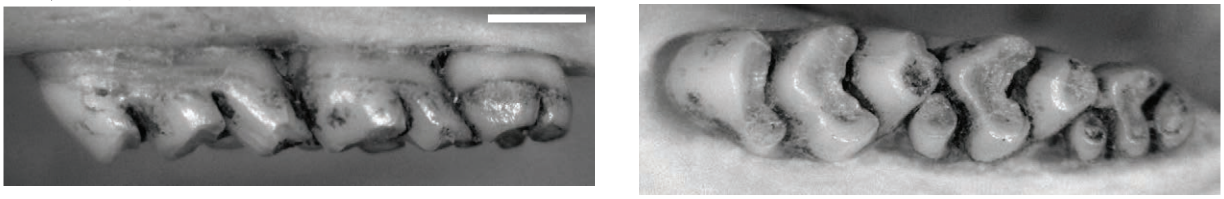

Apodemini

Apodemus agrarius, MCZ 7167

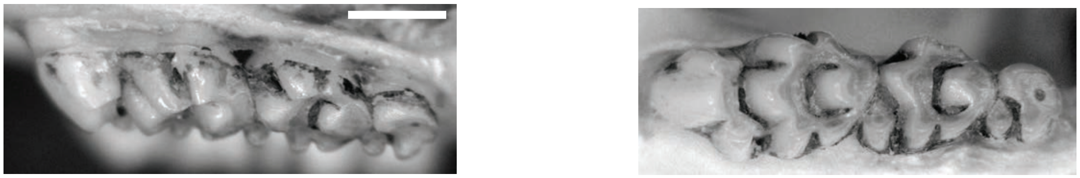

Apodemus flavicollis, USNM 124398

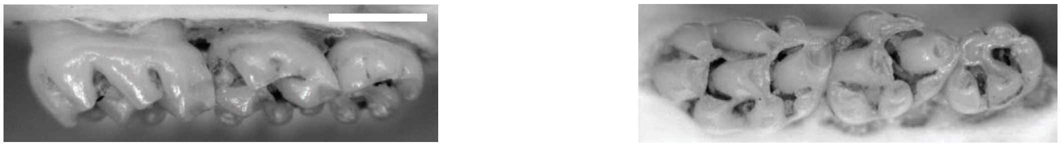

Tokudaia muenninki, USNM 278762

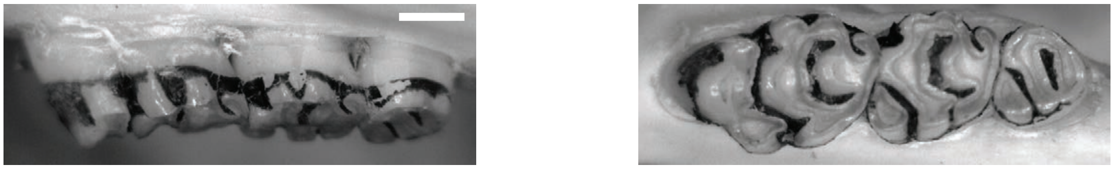

Murini

Mus musculus, MCZ 63120

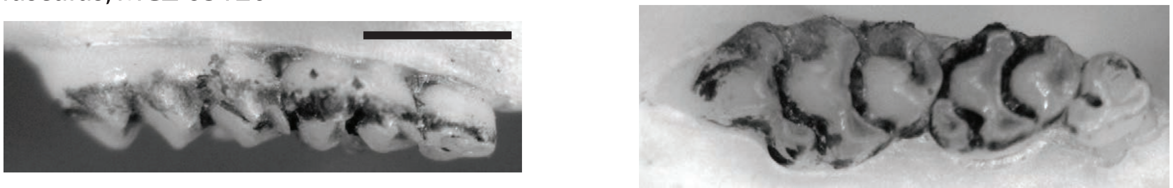

Praomyini

Colomys goslingi, MCZ 17644

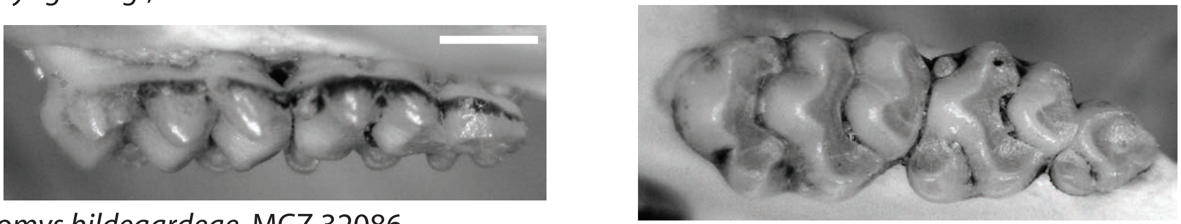

Zelotomys hildegardeae, MCZ 32086

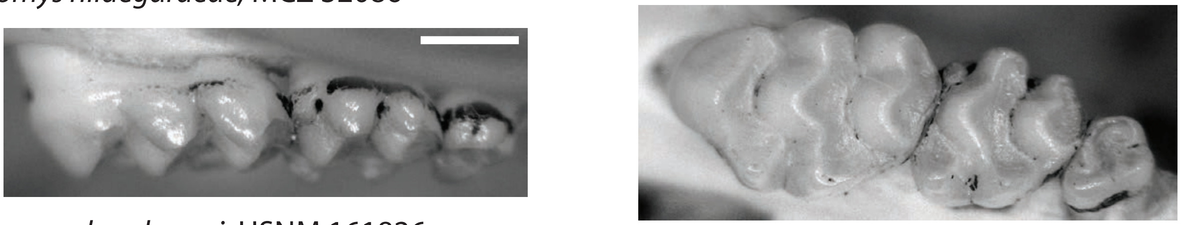

Myomyscus brockmani, USNM 161826

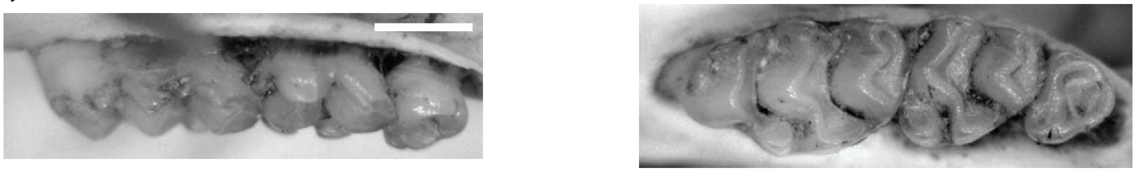

Praomys jacksoni, MCZ31393

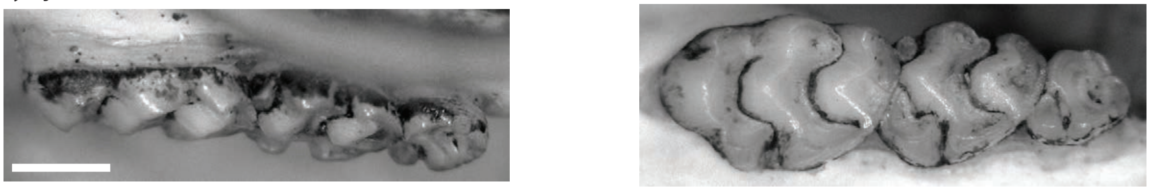

Arvicanthini

Golunda ellioti, MCZ 14883

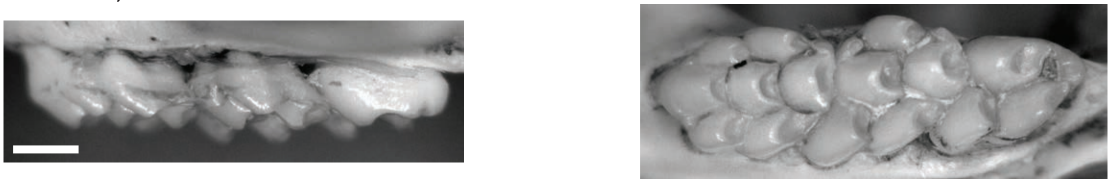

Oenomys hypoxanthus, MCZ 30266

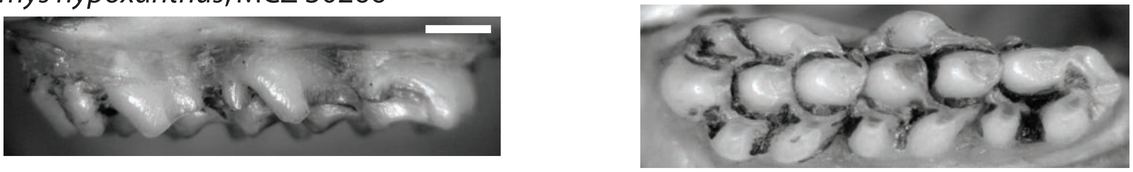

Desmomys harringtoni, USNM 516129

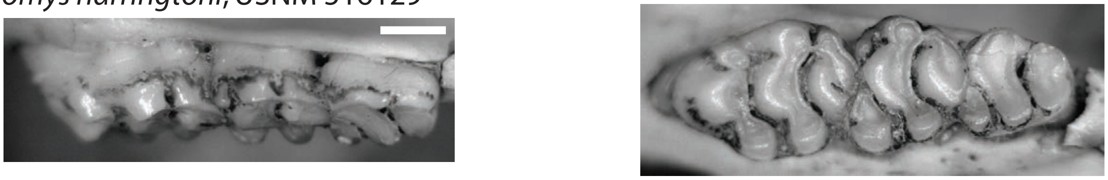

Aethomys kaiseri, USNM 183388

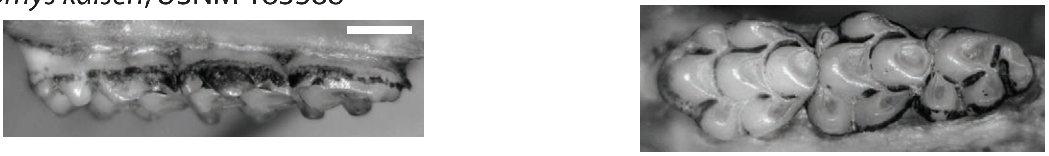

Rhabdomys pumilio, MCZ 26591

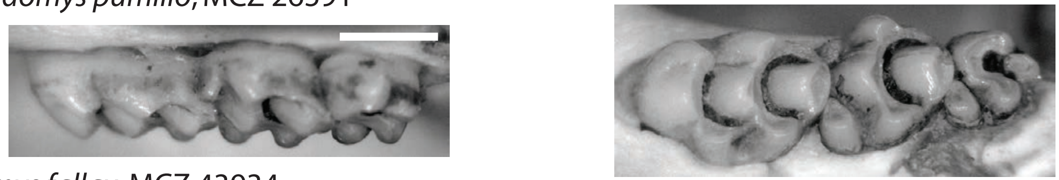

Pelomys fallax, MCZ 43934

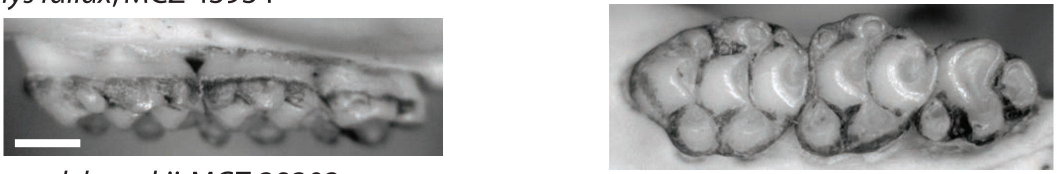

Mylomys dybowskii, MCZ 38303

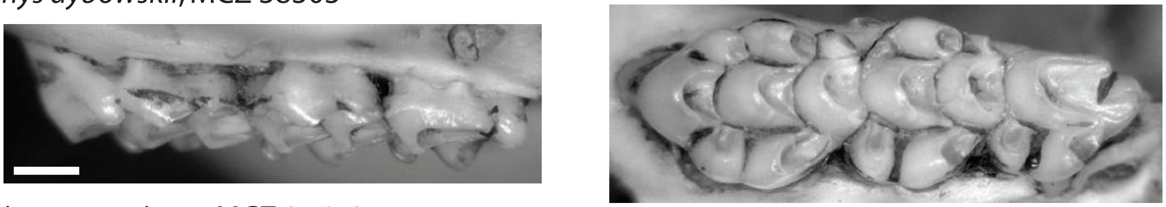

Lemniscomys striatus, MCZ 31673

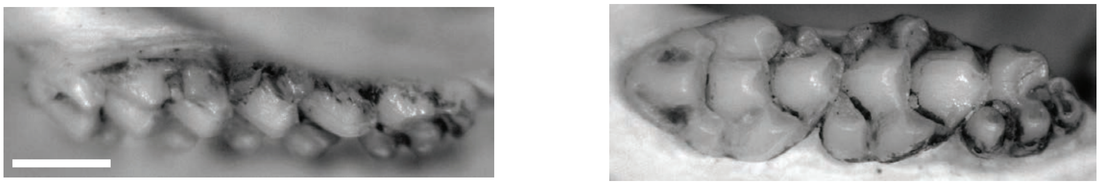

Arvicanthis niloticus, MCZ 31317

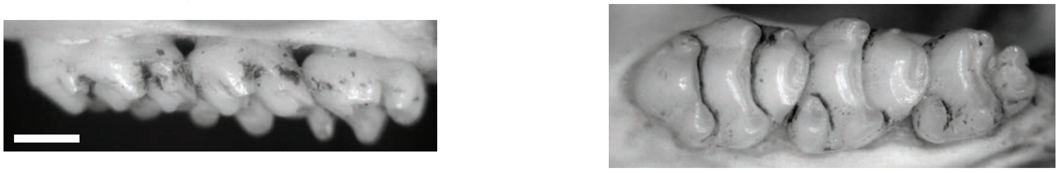

Arvicanthini (*Continued*)

Micaelamys namaquensis, MCZ 36961

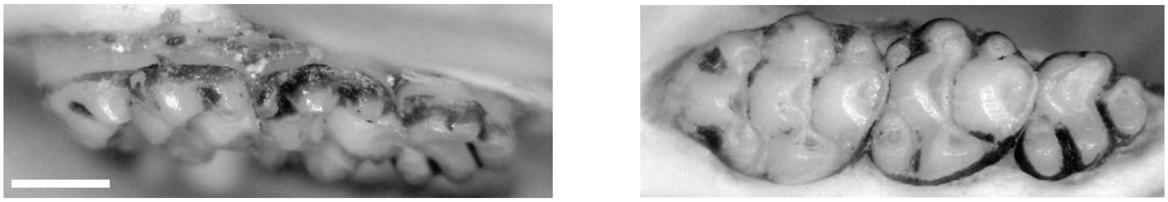

Grammomys macmillani, MCZ 24046

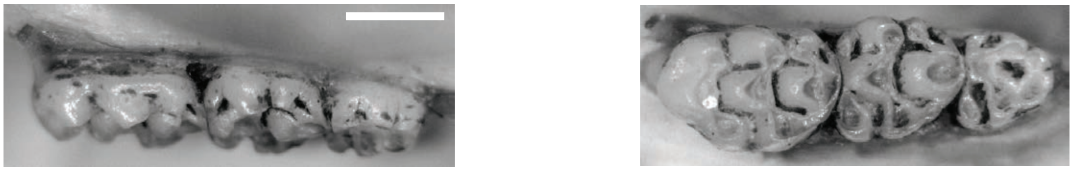

Grammomys dolichurus, MCZ 26661

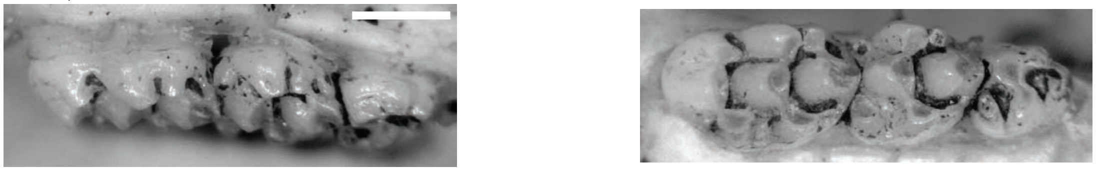

Dasymys incomtus, MCZ 31362

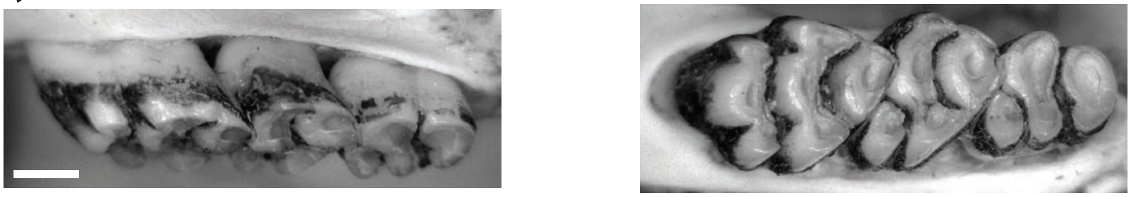

Hybomys univittatus, MCZ 14707

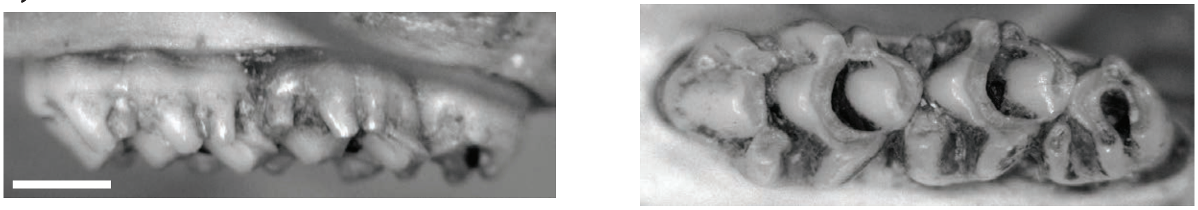

Thallomys nigricauda, USNM 380130

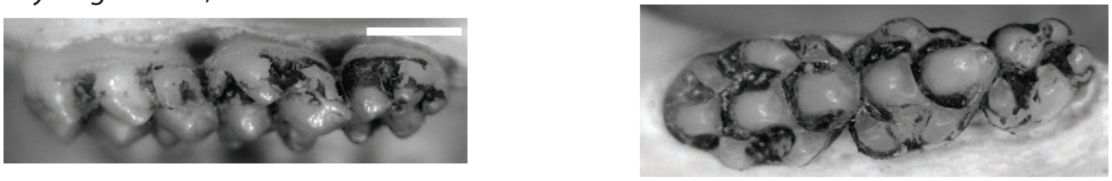

Thamnomys venustus, USNM 340822

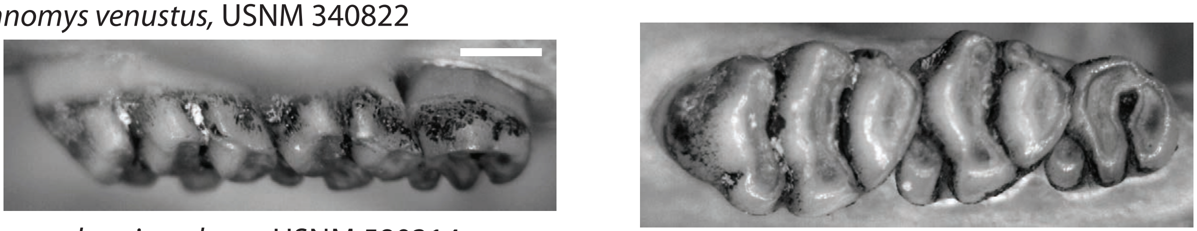

Stochomys longicaudatus, USNM 580314

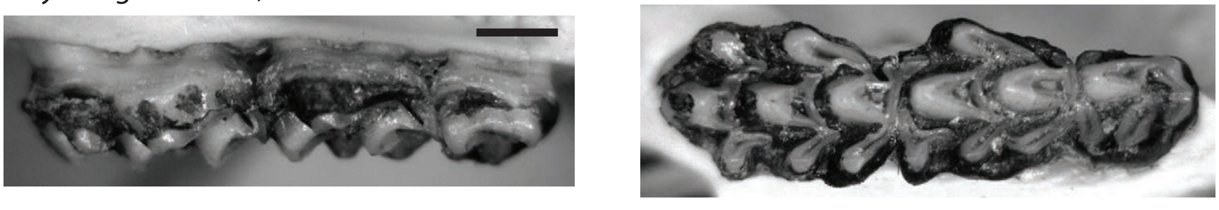

Millardini

Millardia meltada, MCZ14884

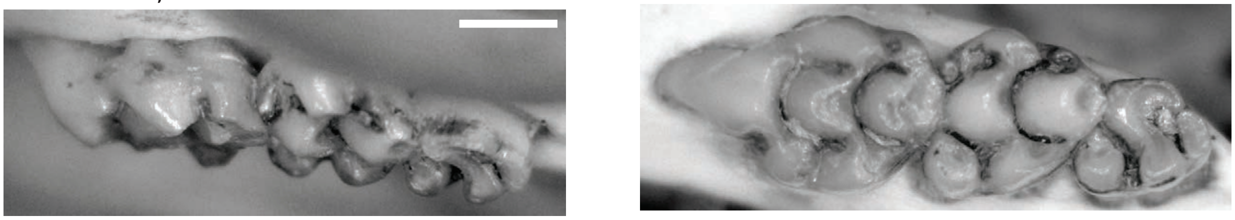

Otomyini

Parotomys littledalei, MCZ 37122

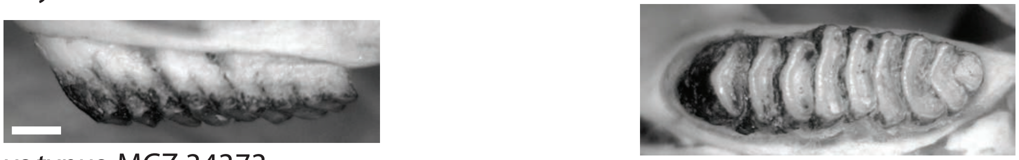

Otomys typus, MCZ 34373

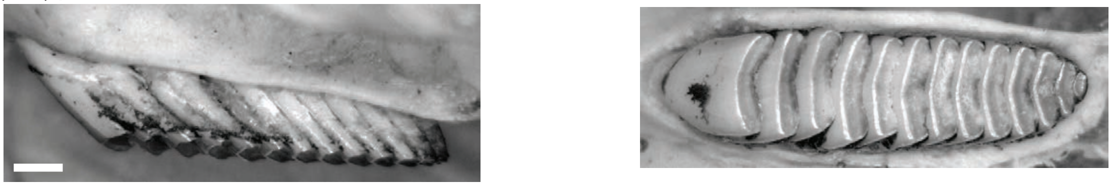

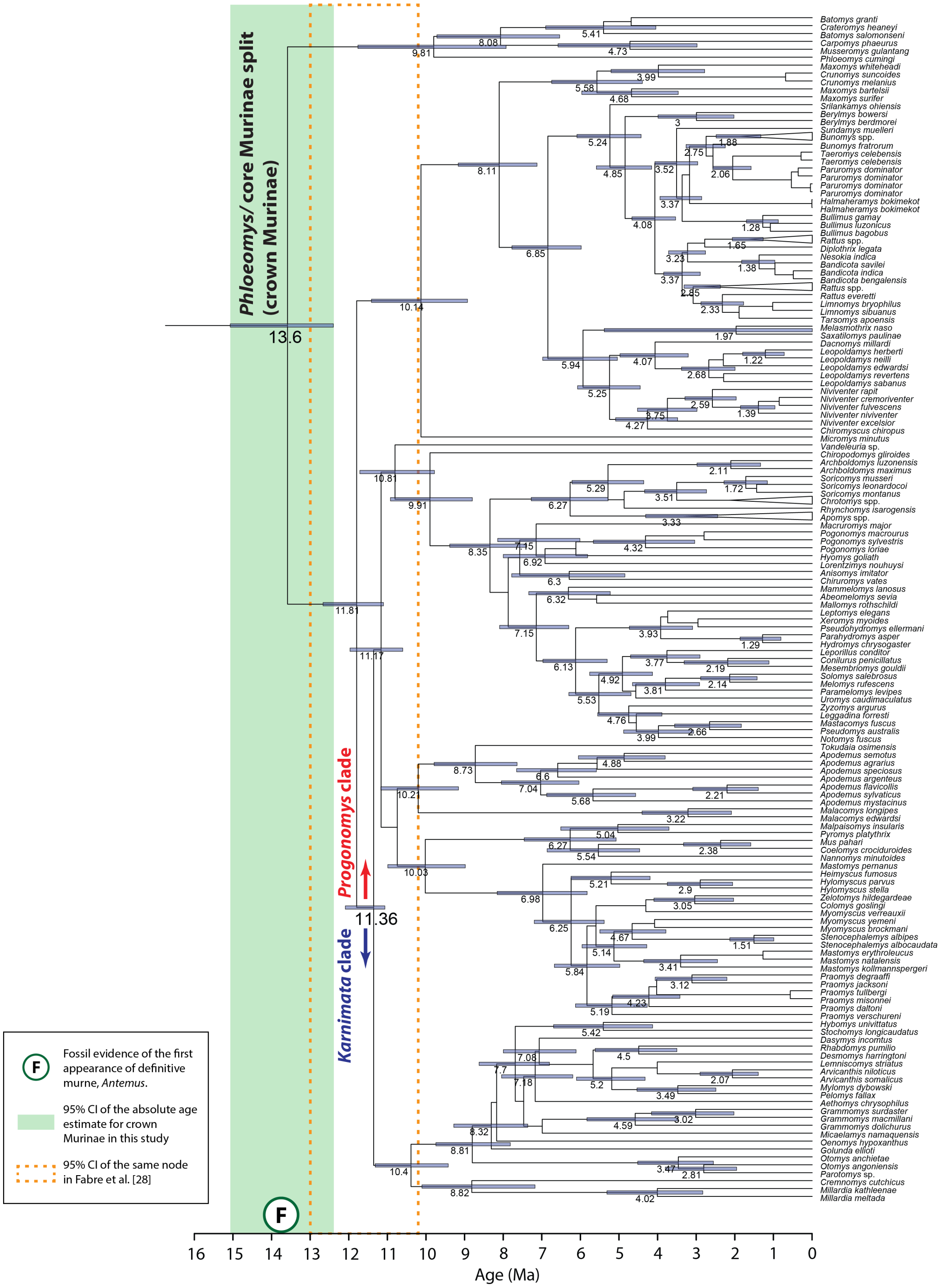

Figure S4. Maximum clade probability tree from the BEAST analysis of the Fabre et al.[29] data using the 11.2 Ma calibration point for the *Mus*/*Arvicanthis* split. Node bars indicate the 95% credible interval of the posterior density of divergence times. Numbers on the node represent the posterior mean of divergence times. A high-resolution image of this figure is available below.

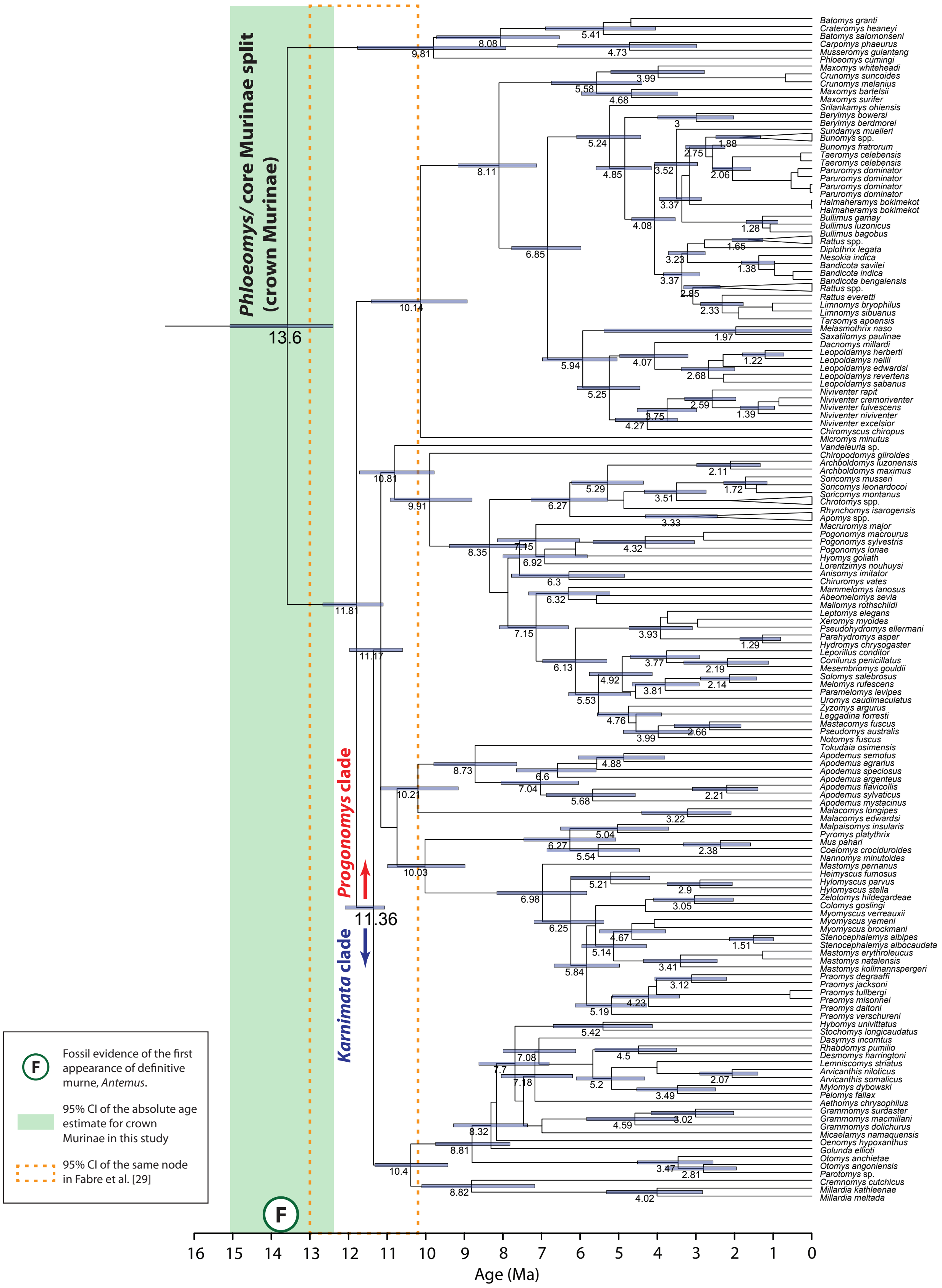

Phloeomys/core Murinae split
(crown Murinae)
13.6
Progonomys clade
Karnimata clade
11.36
Batomys granti
Crateromys heaneyi
Batomys salomonseni
Carpomys phaeurus
Musseromys gulantang
Phloeomys cumingi
Maxomys whiteheadi
Crunomys suncoides
Crunomys melanius
Maxomys bartelsii
Maxomys surifer
Srilankamys ohiensis
Berylmys bowersi
Berylmys berdmorei
Sundamys muelleri
Bunomys spp.
Bunomys fratrorum
Taeromys celebensis
Taeromys celebensis
Paruromys dominator
Paruromys dominator
Paruromys dominator
Paruromys dominator
Halmaheramys bokimekot
Halmaheramys bokimekot
Bullimus gamay
Bullimus luzonicus
Bullimus bagobus
Rattus spp.
Diplothrix legata
Nesokia indica
Bandicota savilei
Bandicota indica
Bandicota bengalensis
Rattus spp.
Rattus everetti
Limnomys bryophilus
Limnomys sibuanus
Tarsomys apoensis
Melasmothrix naso
Saxatilomys paulinae
Dacnomys millardi
Leopoldamys herberti
Leopoldamys neilli
Leopoldamys edwardsi
Leopoldamys revertens
Leopoldamys sabanus
Niviventer rapit
Niviventer cremoriventer
Niviventer fulvescens
Niviventer niviventer
Niviventer excelsior
Chiromyscus chiropus
Micromys minutus
Vandeleuria sp.
Chiropodomys gliroides
Archboldomys luzonensis
Archboldomys maximus
Soricomys musseri
Soricomys leonardocoi
Soricomys montanus
Chrotomys spp.
Rhynchomys isarogensis
Apomys spp.
Macruromys major
Pogonomys macrourus
Pogonomys sylvestris
Pogonomys loriae
Hyomys goliath
Lorentzimys nouhuysi
Anisomys imitator
Chiruromys vates
Mammelomys lanosus
Abeomelomys sevia
Mallomys rothschildi
Leptomys elegans
Xeromys myoides
Pseudohydromys ellermani
Parahydromys asper
Hydromys chrysogaster
Leporillus conditor
Conilurus penicillatus
Mesembriomys gouldii
Solomys salebrosus
Melomys rufescens
Paramelomys levipes
Uromys caudimaculatus
Zyzomys argurus
Leggadina forresti
Mastacomys fuscus
Pseudomys australis
Notomys fuscus
Tokudaia osimensis
Apodemus semotus
Apodemus agrarius
Apodemus speciosus
Apodemus argenteus
Apodemus flavicollis
Apodemus sylvaticus
Apodemus mystacinus
Malacomys longipes
Malacomys edwardsi
Malpaisomys insularis
Pyromys platythrix
Mus pahari
Coelomys crociduroides
Nannomys minutoides
Mastomys pernanus
Heimyscus fumosus
Hylomyscus parvus
Hylomyscus stella
Zelotomys hildegardeae
Colomys goslingi
Myomyscus verreauxii
Myomyscus yemeni
Myomyscus brockmani
Stenocephalemys albipes
Stenocephalemys albocaudata
Mastomys erythroleucus
Mastomys natalensis
Mastomys kollmannspergeri
Praomys degraaffi
Praomys jacksoni
Praomys tullbergi
Praomys misonnei
Praomys daltoni
Praomys verschureni
Hybomys univittatus
Stochomys longicaudatus
Dasymys incomtus
Rhabdomys pumilio
Desmomys harringtoni
Lemniscomys striatus
Arvicanthis niloticus
Arvicanthis somalicus
Mylomys dybowskii
Pelomys fallax
Aethomys chrysophilus
Grammomys surdaster
Grammomys macmillani
Grammomys dolichurus
Micaelamys namaquensis
Oenomys hypoxanthus
Golunda ellioti
Otomys anchietae
Otomys angoniensis
Parotomys sp.
Cremnomys cutchicus
Millardia kathleenae
Millardia meltada
16 15 14 13 12 11 10 9 8 7 6 5 4 3 2 1 0
Age (Ma)
F
Fossil evidence of the first appearance of definitive murne, Antemus.
95% CI of the absolute age estimate for crown Murinae in this study
95% CI of the same node in Fabre et al. [29]
